# Genes *ptz* and *idi*, Coding for Cytokinin Biosynthesis Enzymes, Are Essential for Tumorigenesis and *In Planta* Growth by *P. syringae* pv. savastanoi NCPPB 3335

**DOI:** 10.3389/fpls.2020.01294

**Published:** 2020-08-21

**Authors:** Maite Añorga, Adrián Pintado, Cayo Ramos, Nuria De Diego, Lydia Ugena, Ondřej Novák, Jesús Murillo

**Affiliations:** ^1^ Institute for Multidisciplinary Research in Applied Biology, Universidad Pública de Navarra, Mutilva Baja, Spain; ^2^ Área de Genética, Facultad de Ciencias, Universidad de Málaga, Málaga, Spain; ^3^ Instituto de Hortofruticultura Subtropical y Mediterránea “La Mayora”, Consejo Superior de Investigaciones Científicas (IHSM-UMA-CSIC), Málaga, Spain; ^4^ Department of Chemical Biology and Genetics, Centre of the Region Haná for Biotechnological and Agricultural Research, Faculty of Science, Palacký University, Olomouc, Czechia; ^5^ Laboratory of Growth Regulators, Faculty of Science, Palacký University, Olomouc, Czechia; ^6^ Institute of Experimental Botany, Czech Academy of Sciences, Olomouc, Czechia

**Keywords:** *Pseudomonas savastanoi* pv. savastanoi, olive knot, tumor, pathogenicity, virulence genes, virulence plasmids, indoleacetic acid, phytohormones

## Abstract

The phytopathogenic bacterium *Pseudomonas syringae* pv. savastanoi elicits aerial tumors on olive plants and is also able to synthesize large amounts of auxins and cytokinins. The auxin indoleacetic acid was shown to be required for tumorigenesis, but there is only correlational evidence suggesting a role for cytokinins. The model strain NCPPB 3335 contains two plasmid-borne genes coding for cytokinin biosynthesis enzymes: *ptz*, for an isopentenyl transferase and *idi*, for an isopentenyl-diphosphate delta-isomerase. Phylogenetic analyses showed that carriage of *ptz* and *idi* is not strictly associated with tumorigenic bacteria, that both genes were linked when first acquired by *P. syringae*, and that a different allele of *ptz* has been independently acquired by *P. syringae* pv. savastanoi and closely related bacteria. We generated mutant derivatives of NCPPB 3335 cured of virulence plasmids or with site-specific deletions of genes *ptz* and/or *idi* and evaluated their virulence in lignified and micropropagated olive plants. Strains lacking *ptz*, *idi*, or both produced tumors with average volumes up to 29 times smaller and reached populations up to two orders of magnitude lower than those induced by strain NCPPB 3335; these phenotypes reverted by complementation with the cloned genes. *Trans*-zeatin was the most abundant cytokinin in culture filtrates of NCPPB 3335. Deletion of gene *ptz* abolished biosynthesis of *trans*-zeatin and dihydrozeatin, whereas a reduced but significant amount of isopentenyladenine was still detected in the medium, suggesting the existence of other genes contributing to cytokinin biosynthesis in *P. syringae*. Conversely, extracts from strains lacking gene *idi* contained significantly higher amounts of *trans*-zeatin than extracts from the wild-type strain but similar amounts of the other cytokinins. This suggests that Idi might promote tumorigenesis by ensuring the biosynthesis of the most active cytokinin forms, their correct balance *in planta*, or by regulating the expression of other virulence genes. Therefore, gene *ptz*, but not gene *idi*, is essential for the biosynthesis of high amounts of cytokinins in culture; however, both *ptz* and *idi* are individually essential for the adequate development of tumors on olive plants by Psv NCPPB 3335.

## Introduction

Plant defense against offenders is expensive, requiring an investment that could otherwise be used for reproduction. Plants have therefore evolved resource management mechanisms diverting energy to either growth or defense, depending on environmental conditions: the ‘growth–defense trade-off’ phenomenon (Huot et al., 2014). These adaptive responses are largely controlled by a delicate balance of phytohormones, and so it is not surprising that phytopathogens also evolved a myriad of sophisticated strategies to interfere with this balance to facilitate pathogen colonization and disease development (Denancé et al., 2013; Ma and Ma, 2016).

The gammaproteobacterium *Pseudomonas* *syringae* is a complex of taxonomically diverse species that cause economically relevant diseases in many annual and woody plants (Lamichhane et al., 2014; Lamichhane et al., 2015). This bacterial complex comprises at least 13 phylogroups (PGs), traditionally distributed into more than 60 pathovars, which are infrasubspecific groups defined by their ability to cause distinctive disease syndromes in a set of defined plant hosts (Young, 2010). Pathogenicity and virulence are generally dependent on a type III secretion system (T3SS), delivering pathovar- and strain-specific suites of effector proteins into the cell cytoplasm, mainly to suppress plant defense responses (Cunnac et al., 2009; Dillon et al., 2019a). Nevertheless, *P. syringae* strains produce a diversity of other molecules that increase virulence or microbial fitness, among which phytotoxins and hormones are paramount for their relevance and wide distribution (Glickmann et al., 1998; Bender et al., 1999; Cunnac et al., 2009; Caballo-Ponce et al., 2017a; Dillon et al., 2019b). *P. syringae* commonly induces spots and blights of leaves, stems and fruits, with bacterial cankers and blights as the most common diseases of woody plants (Agrios, 2005; Lamichhane et al., 2014). Another frequent type of symptoms induced in woody hosts are galls or tumors (Lamichhane et al., 2014; Caballo-Ponce et al., 2017a), which are exclusively caused by *P. syringae* pathovars cerasicola, daphniphylli, dendropanacis, myricae, nerii, retacarpa, rhaphiolepidis, and savastanoi plus *P. meliae* and *P. tremae*, all belonging to PG3 (also called genomospecies 2; syn. *P. amygdali*) (Gardan et al., 1999; Gomila et al., 2017).

*P. syringae* pv. savastanoi (Psv) causes tumors in olive (*Olea europaea*) and is a prominent research model for woody pathosystems (Ramos et al., 2012; Caballo-Ponce et al., 2017a). As occurs with pathovars infecting annual plants, pathogenicity of Psv is dependent on the functionality of a type III secretion system (Sisto et al., 2004; Pérez-Martínez et al., 2010). Additionally, the virulence of Psv is modulated by several other factors, including Na^+^/Ca^2+^ exchange (Moretti et al., 2019), quorum sensing, metabolism of phenolics, c-di-GMP metabolism, and, importantly, the production of the phytohormones indoleacetic acid (IAA) and cytokinins (CKs) (Caballo-Ponce et al., 2017a). The biosynthesis of high levels of IAA in *P. syringae* takes place through the indole-3-acetamide pathway and requires the *iaaMH* operon (Comai and Kosuge, 1980; Yamada et al., 1985; Aragón et al., 2014), which has a rather restricted distribution within *P. syringae* (Glickmann et al., 1998; Ramos et al., 2012). Genes *iaaMH* have a large and complex contribution to virulence and are required for the elicitation of tumors on olive plants; they increase *in planta* competitiveness and also affect the expression of other virulence-related genes (Silverstone et al., 1993; Aragón et al., 2014).

**Figure 1:**
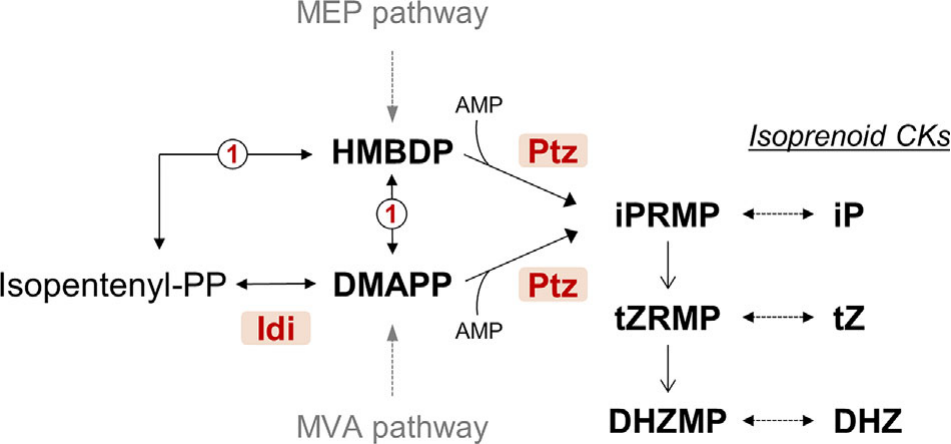
Simplified predicted pathway for the biosynthesis of cytokinins by *Pseudomonas syringae* pv. savastanoi NCPPB 3335. CKs, cytokinins; DHZMP, dihydrozeatin riboside monophosphate; DHZ, dihydrozeatin; DMAPP, dimethylallyl diphosphate; Idi, isopentenyl-diphosphate delta-isomerase (locus_tag PSPSV_C0024); HMBDP, hydroxymethylbutenyl diphosphate; iP, isopentenyladenine; iPRMP, isopentenyladenosine 5′-monophosphate; Ptz, isopentenyl transferase (locus_tag PSPSV_A0024); tZ, *trans*-zeatin; tZRMP, *trans*-zeatin riboside 5′-monophosphate. MEP and MVA are the methylerythritol phosphate and the mevalonate pathways, respectively. Enzyme marked as 1 in a circle is the predicted product of gene PSA3335_RS23935, annotated as 4-hydroxy-3-methylbut-2-enyl diphosphate reductase. Dotted arrows indicate more than one reaction step. The pathway is condensed from the Kyoto Encyclopedia of Genes and Genomes (KEGG) database and published pathways (Sakakibara, 2006; Sakakibara, 2010).

Natural CKs are substituted adenine molecules carrying on the *N^6^* position an aromatic or, more frequently, an isoprenoid-derived side chain (Sakakibara, 2006; Sakakibara, 2010). Biosynthesis of isoprenoid CKs can follow two possible pathways. One of the pathways is derived from tRNA degradation and leads to *cis*-zeatin (cZ)-type CKs, which have not been detected in cultures of tumorigenic *P. syringae* pathovars. The second pathway derives from isopentenylation of free adenine nucleotides. This is initiated by the condensation of AMP with dimethylallyldiphosphate (DMAPP) or of hydroxylated derivatives of DMAPP such as hydroxymethylbutenyl diphosphate (HMBDP) and is catalyzed by the key enzyme, isopentenyl transferase (**Figure 1**). CKs synthesized through this pathway were detected in cultures of *P. syringae* pathovars savastanoi and nerii, the latter highly related to Psv and also producing tumors on oleander, as well as in *P. amygdali*. Diverse works report the detection in these bacteria of up to eight types of CKs (*trans*-zeatin (tZ), dihydrozeatin (DHZ), *trans*-zeatin riboside (tZR), dihydrozeatin riboside (DHZR), isopentenyl adenine (iP), isopentenyl adenosine (iPR), 1′-methylzeatin and ribosyl-1″-methylzeatin), although there is considerable variability in the types and amount of CKs produced by different strains (Surico et al., 1975; Surico et al., 1985a; Evidente et al., 1986; MacDonald et al., 1986; Iacobellis et al., 1990; Morris et al., 1991; Marchi et al., 2011). A gene for isopentenyl transferase (gene *ptz*) has been cloned from Psv and shown to direct the biosynthesis of certain CKs in culture when expressed in *E. coli* (MacDonald et al., 1986; Powell and Morris, 1986). Gene *idi* (originally called *ipt*), coding for an isopentenyl-diphosphate delta-isomerase, has also been found in Psv (Bardaji et al., 2011). Its product is predicted to provide the substrate DMAPP for the Ptz enzyme (**Figure 1**), but its role in the biosynthesis of CKs has not been demonstrated.

Genes for biosynthesis of phytohormones by Psv and *P. syringae* pv. nerii are sometimes located in native plasmids, and hence, it was shown that plasmid curing can lead to a reduction in the size of tumors (MacDonald et al., 1986; Iacobellis et al., 1994; Pérez-Martínez et al., 2008; Bardaji et al., 2011). Nevertheless, plasmid curing could completely abolish or diminish CK production, indicating that more than one gene could participate in CK biosynthesis. Diverse studies have also found a positive association between the amount of CKs naturally produced by certain *P. syringae* strains and the size of knots induced in the plant host (Surico et al., 1985b; Cinelli et al., 2014; Schiff et al., 2019), although this is not always true, suggesting the existence of additional strain-specific genes contributing to virulence. Similarly, a strain of *P. syringae* pv. nerii was able to induce tumors of nearly wild-type size in oleander, despite being unable to synthesize CKs (Roberto and Kosuge, 1987). Therefore, and taking into account the plethora of virulence genes carried by native plasmids of the *P. syringae* complex (Vivian et al., 2001; Sundin, 2007), and that knock-out mutants of CKs-related genes have not yet been reported, these correlational studies do not clarify the impact of Psv-produced CKs in virulence.

The model strain Psv NCPPB 3335 carries genes *ptz* and *idi* in plasmids pPsv48A and pPsv48C, respectively (Bardaji et al., 2011). In this work, we obtained a plasmidless derivative of NCPPB 3335 as a tool for the identification of virulence genes and, together with the generation of site-directed mutants of genes *ptz* and *idi*, to evaluate the role of CKs in its pathogenicity in olive plants. We demonstrate that gene *ptz* is essential, but not gene *idi*, for the biosynthesis of CKs in culture and that both genes and/or their products are required for the development of tumors by Psv NCPPB 3335.

## Material and Methods

### Bacterial Strains and Growth Conditions

Bacterial strains and plasmids are detailed in **Supplementary Table S1**. Strains were routinely propagated at 25°C or 37°C, for *P. syringae* and *E. coli*, respectively, using LB (Sambrook et al., 1989) or nutrient agar (NA; Oxoid), which was supplemented with 5% w/v of sucrose (medium SNA) for counter selection of clones containing Tn*5*-GDYN1. When necessary, media were supplemented with the following antibiotics (final concentrations in µg ml^−1^ for *Pseudomonas*/*E. coli*): ampicillin (Amp) 300/100, gentamicin (Gm) 12.5, kanamycin (Km) 7/50, nitrofurantoin (Nf) 100, spectinomycin (Spc) 25, tetracycline (Tc) 12.5.

### Molecular Techniques and Bioinformatics

General molecular biology techniques were followed (Sambrook et al., 1989). Plasmid DNA from *Pseudomonas* was purified by a rapid alkaline lysis (Zhou et al., 1990); plasmids from *E. coli* were purified using a boiling lysis method (Holmes and Quigley, 1981) or a commercial kit (Illustra plasmid Prep Mini Spin Kit, GE Healthcare). Undigested plasmids were separated in 0.8% agarose gels by electrophoresis (Sesma et al., 2000). Plasmids were transferred to *P. syringae* by electroporation (Choi et al., 2006).

PCR amplifications were done using standard (BIOTaq, Bioline, UK) or high-fidelity (PrimeStar HS, Takara Bio Inc., Japan) polymerase mixes and using primers listed in **Supplementary Table S2**. Amplicons were purified using the QIAquick PCR Purification Kit (QIAGEN) and cloned into vectors pJET1.2 (CloneJET PCR Cloning Kit; Thermo Scientific) or pGEM-T Easy (Promega). Genomic DNA was purified using the Jet Flex Extraction (Genomed; Löhne, Germany) kit. Sanger and whole genome sequencing using Illumina Myseq 2 × 300 were done by Macrogen Inc. (Amsterdam). Paired reads (coverage 60×) from strain UPN912 (ΔABC) were qualitatively assessed before *de novo* assembling with CLC Genomics Workbench (v 10.1.1) software (BioProject PRJNA638121). Sequences were visualized and manipulated using the Artemis and ACT software (Carver et al., 2008). Sequence comparisons were done using the Blast algorithms (Hubbard et al., 2008) and sequence alignments using the web servers for MultAlin (Corpet, 1988) or for EMBL-EBI tools (Chojnacki et al., 2017). Promoters were predicted using the online version of BProm (Softberry Inc., Mt. Kisco, NY; Solovyev and Salamov, 2011). Primers were designed with Primer3plus (Untergasser et al., 2012). Sequence alignments using Muscle, substitution model selection, phylogenetic analyses using the Maximum Likelihood method and branch support for the resulting trees by bootstrap analysis with 200 replicates were done using MEGA7 (Kumar et al., 2016).

Genes *ptz* and *idi* were deleted from pPsv48A and pPsv48C, respectively, by marker exchange as follows. For *ptz* we amplified DNA fragments of approximately 1 kb, corresponding to the upstream and downstream flanking regions, with primers FAnewptz-F/R and FP_ptz-F18/FP_ptz-R-694 (**Supplementary Table S2**). Restriction sites for BamHI were included in the primers as described previously (Matas et al., 2014). The resulting product, consisting of upstream and downstream flanking regions separated by a BamHI restriction site, was cloned into pGEMT (Amp^R^), and the Km resistance gene *nptII* from pGEM-T-KmFRT-BamHI was cloned into the BamHI site. The plasmid was transferred to Psv NCPPB 3335 by electroporation (Pérez-Martínez et al., 2007), and transformants were selected on LB-Km plates. To select the allelic exchange, individual colonies were replicated onto LB-Amp plates and Amp^R^ colonies were discarded. For gene *idi*, a 3,394 nt fragment spanning *idi* was amplified with primers mutipt_F/R (**Supplementary Table S2**) and cloned in pJET1.2. This construct was digested with SpeI and SmaI, liberating a 753 nt fragment spanning all of gene *idi* plus the last 157 nt of the previous CDS (PSPSV_C0023), ligated to the Spc^R^ cassette amplified from pHP45Ω and transferred to strain Psv48ΔAB and selecting on LB plus Spc. Clones were then serially transferred six consecutive times on LB to select, at the end of these transfers, a Spc^R^ and Amp^S^ clone, designated UPN1020 and containing pPsv48CΔidi. The double mutant *ptz^-^idi^-^* was constructed by transferring pPsv48CΔidi to strain Δptz (UPN1028) and selecting on LB plus Spc and Km. All mutations were confirmed by PCR, Southern blot hybridization, and/or sequencing.

We constructed diverse plasmid for complementation *in trans* of the different mutants. The complete gene *ptz*, including the CDS, its promoter, and transcriptional terminator, was amplified by PCR as a 1,372 nt fragment using primers ExpptZ-F1/R2 (**Supplementary Table S2**) and cloned into pGEM-T. The fragment was then liberated with EcoRI and subcloned into pBBR1MCS-5; a clone containing *ptz* oriented for transcription from the constitutive P*_lac_* promoter of the vector was designated pIPMptz and selected for complementation analyses. Gene *idi* was amplified as a 1,117 nt fragment containing its predicted promoter, with primers iptC_F/R (1,117 nt) (**Supplementary Table S2**), or as a 2,311 nt fragment also containing the upstream CDS PSPSV_C0023, using primers iptC_R/iptSAM_F (**Supplementary Table S2**, **Supplementary Figure S1**) and cloned in pJET1.2. The inserts from the resulting constructions were liberated with BglII and individually cloned in the same site behind a T4 transcription terminator either in pME6031, for the 1.1 kb fragment and generating p31idi, or in pME6041, for the 2.3 kb fragment and generating p41idiSAM. The cloned 1,372 nt fragment of gene *ptz* was amplified using primers ptz_F1/R1 (**Supplementary Table S2**), containing EcoRI adapters and cloned in pJET1.2. The EcoRI insert was liberated and cloned in p31idi to yield p31idiptz. All constructions were confirmed by sequencing.

### Curing of Plasmid pPsv48C to Yield ΔABC

Strain ΔAB is cured of plasmids pPsv48A and pPsv48B but could not be cured of plasmid C (Bardaji et al., 2011). Therefore, this strain was mutagenized using transposon Tn*5-*GDYN1, essentially as described (Flores et al., 1993; Bardaji et al., 2011). This transposon carries a *sacB* gene conferring lethality in the presence of 5% sucrose and facilitates the selection of strains cured of native plasmids. One of the resulting mutants contained an insertion in pPsv48C after position 7,433, which was mapped as described (Bardaji et al., 2019) and was retained as strain UPN877. Plasmid pPsv48C carries two functional replicons (Bardaji et al., 2017) and three functional toxin–antitoxin modules that provide a very high stability (Bardaji et al., 2019). To facilitate curing of pPsv48C from UPN877, we functionally inactivated these toxin–antitoxin modules using the cloned antitoxins in plasmid pRK3C as described (Bardaji et al., 2019). For plasmid curing, overnight cultures of strain UPN877(pRK3C) on LB were serially diluted and spread on SNA plates and incubated at 25°C. The plasmid profile of well-separated colonies was then examined for the loss of pPsv48C::Tn*5*-GDYN1, and selection of the plasmidless strain ΔABC (UPN912) was done as described in *Results*.

### Quantification of Cytokinins

Bacterial strains were grown overnight at 25°C with shaking (200 rpm) in minimal A medium (in g/l, K_2_HPO_4_, 10.5; KH_2_PO_4_, 4.5; (NH_4_)_2_SO_4_, 1; Na-citrate 2 H_2_O, 0.5; MgSO_4_, 0.12)(Miller, 1992) supplemented with casamino acids and glucose at final concentration of 0.04 and 2 g/l, respectively (MacDonald et al., 1986). These were used to inoculate fresh minimal A medium to a OD_600_ of 0.1 and grown in the same conditions until reaching an OD_600_ of 0.6, after approximately 12–14 h. Cell-free culture supernatants were then collected by centrifugation and filtration through 2 μm filters, and immediately frozen in liquid nitrogen until analyzed. For CK analyses we used three independent cultures, with two samples per culture, for each strain. A total of 200 µl from each culture was collected twice and used as biological replicate for the cytokinin analysis, ending with 4 replicates per bacterial strain. Additionally, each replicate was extracted and quantified twice (technical replicate). Briefly, each culture was extracted using ice-cold modified Bieleski buffer (methanol/water/formic acid, 15/4/1, v/v/v) containing isotope-labelled CK internal standards (0.5 pmol of CK bases, ribosides, N-glucosides, 1 pmol of CK O-glucosides and nucleotides). The extract was then purified using two solid phase extraction columns, the octadecylsilica-based column (C18, 500 mg of sorbent, Applied Separations) and the Oasis MCX column (30 mg/1 ml, Waters), according to the method described by (Dobrev and Kaminek, 2002), including modifications described by Antoniadi et al. (2015).

### Pathogenicity Assays

Micropropagated olive (*Olea* *europaea*) plantlets derived from a seed germinated *in vitro*, originally collected from a cv. Arbequina plant, were propagated and rooted in DKW medium (Driver and Kuniyuki, 1984) essentially as described (Rodríguez-Moreno et al., 2008). Rooted plantlets were transferred to DKW medium without hormones, and maintained in a growth chamber with a 16/8 h light/dark photoperiod, light intensity of 35 µmoles m^−2^ s^−1^ and 25°C for at least two weeks; then, inoculations were done in sterility when plantlets were 60–80 mm long and had 3–5 internodes. All strains used as inocula contained plasmid pLRM1-GFP (Gm^R^) (Rodríguez-Moreno et al., 2009), expressing GFP, to monitor tumor colonization by epifluorescence microscopy. Bacteria used for inoculations were scrapped off LB plates grown for 2 days, washed twice in 10 mM MgCl_2_ and resuspended in this buffer to an OD_600_ of 0.5 (approximately 5 × 10^7^ cfu ml^−1^); then, 10^3^ cfu were applied on a wound made with a scalpel on the stem on each plantlet. Plantlets were then maintained in the growth chamber in the same conditions as above and scored after 28 days using a stereoscopic fluorescence microscope (Leica MZ FLIII; Leica Microsystems, Wetzlar, Germany). Inoculations were done in a similar way for lignified olive plants (at least one-year-old) by depositing 10^6^ cfu in a wound made in the stem, as described (Penyalver et al., 2006; Pérez-Martínez et al., 2007; Matas et al., 2012). Plants were maintained in a greenhouse under natural conditions, with an average temperature of 27°C and 58% of relative humidity, and symptoms were scored after 90 days using a high-resolution camera (Canon D600; Canon Inc., Tokyo, Japan).

Knot volumes developed on micropropagated olive plants were quantified using a 3D scanner and the MINIMAGICS 2.0 software. Weight and volume of tumors developed in one-year old lignified olive plants were obtained as described (Moretti et al., 2008; Hosni et al., 2011). To estimate bacterial populations, knots were crushed and homogenized in 10 mM MgCl_2_ and serial dilutions plated on LB supplemented with appropriate antibiotics for colony counting after two days of growth. Tumor weight and volume, as well as bacterial populations, were averaged from at least three independent replicates.

Means were compared using an analysis of variance (ANOVA) followed, when needed, by Duncan’s multiple range test (p < 0.05). We used software R Project 3.3.3 (R Core Team, 2017) to perform the statistics.

## Results

### Differential Distribution of Genes *ptz* and *idi* Among Tumorigenic Strains of *P. syringae* From PG3 (Genomospecies 2)

Given their potential role in pathogenesis and the elicitation of tumors, we evaluated the distribution and conservation of genes *ptz* and *idi* among the *P. syringae* complex.

BlastP comparisons identified homologs of the deduced products of genes *ptz* and *idi* in a variety of bacteria (**Supplementary Figure S2**). Phylogenetic analyses of these sequences immediately evidenced the separate evolution of these two genes before invading *P. syringae* because the closest relatives of Ptz and Idi are carried by phylogenetically different bacteria. The closest homologs of Ptz are in bacteria interacting with plants, including beneficial interactions, but those of Idi are not, being carried by bacteria exploiting other ecological niches. This dissimilar distribution of genes *ptz* and *idi* suggests that *ptz* is a gene widely required by bacteria for their successful interaction with plants, including the induction of tumors, but that gene *idi* is not.

**Figure 2:**
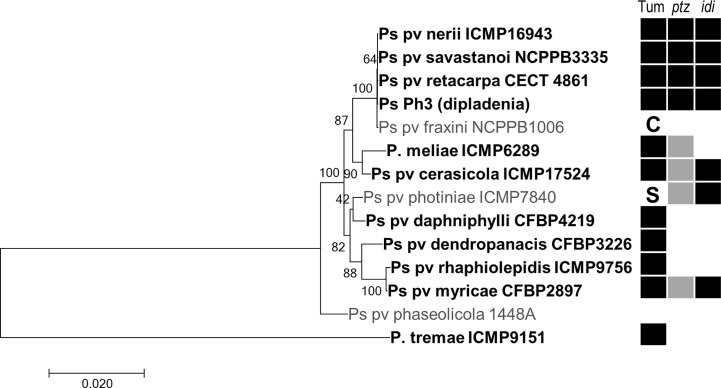
Distribution of cytokinin biosynthesis genes in selected bacteria of the *P.* syringae complex. ML tree based on the concatenated nt sequence of 10 housekeeping genes of representative strains of each pathovar, inferred using the Tamura–Nei model with a discrete gamma distribution with five categories using MEGA 7. Nodes indicate bootstrap percentages over 200 replicas, and branch lengths are the number of substitutions per site. Bacteria reported to induce tumors are indicated in bold and with a black square (Tum); other symptoms: C, cankers and wart-like excrescences; S, leaf spots and blights. Filled and blank squares indicated presence or absence, respectively, of the gene in each pathovar, as assessed by blast and/or PCR. The two colors for gene *ptz* indicate the two distinct homologs found.

Among bacteria of the *P. syringae* complex, we found genes for both Ptz and Idi in the tumorigenic pathovars cerasicola, myricae, nerii, retacarpa, and savastanoi and strain Ph3 from dipladenia (*Mandevilla* spp.) (**Figure 2**). Both genes were also present in *P. syringae* pv. photiniae, which is not reported to induce tumors but produces leaf spots and blights on cherry (Goto, 1983). Additionally, and although they are all reported to be tumorigenic bacteria (Ogimi, 1977; Ogimi et al., 1988a; Ogimi et al., 1988b; Ogimi et al., 1990; Ogimi et al., 1992), *P. meliae* only contained *ptz*, whereas *P. syringae* pvs. daphniphylli, dendropanacis, rhaphiolepidis, and tremae did not carry any of these two genes. The *idi* gene was highly conserved in all these strains, but we found two distinct homologs of gene *ptz* showing a global identity of only around 57% nt/47% aa between them (**Figure 2**, **Supplementary Figure S2**). As occurs in diverse strains of *P. syringae* pvs. nerii and savastanoi (Caponero et al., 1995; Ramos et al., 2012), some of the *ptz* and *idi* homologs were located in plasmids in *P. meliae* and *P. syringae* pvs. cerasicola, myricae, and photiniae (**Supplementary Figure S3**). Using blastp, blastn, and tblastn, we did not find homologs of genes *ptz* and *idi* in any other genome from bacteria of the *P. syringae* complex.

An analysis of the available genomes in the NCBI (March 2020) showed that genes *ptz* and *idi* are located in the same contig in *P. syringae* pv. myricae ICMP 11510, separated by a putative class B radical SAM methyltransferase gene and an insertion of IS*Psy46*, and that this arrangement is conserved in other *P. syringae* pv. myricae strains. The methyltransferase and the *idi* genes were highly similar and syntenic in pPsv48C, from Psv NCPPB 3335. Additionally, in pPsv48C the last 27 nt of *ptz* are found preceding the methyltransferase gene, whereas the remaining *ptz* gene is missing and replaced by a fragment of the mobile element IS*Pa26* (**Supplementary Figure S4**).

Together, these results show that the possession of genes *ptz* and *idi* by plant pathogenic bacteria is not strictly associated with the induction of tumors in the host plant, that both genes were linked when first acquired by PG3 (genomospecies 2), and that a different allele of *ptz* has been independently acquired by the Psv clade (as shown in bold in **Figure 2**). Noticeably, the *ptz* gene from strain NCPPB 3335 is included in a putative genomic island in plasmid A (Bardaji et al., 2011), which might have favored its distribution among the bacterial population.

### Generation of a Plasmidless Derivative of NCPPB 3335

**Figure 3:**
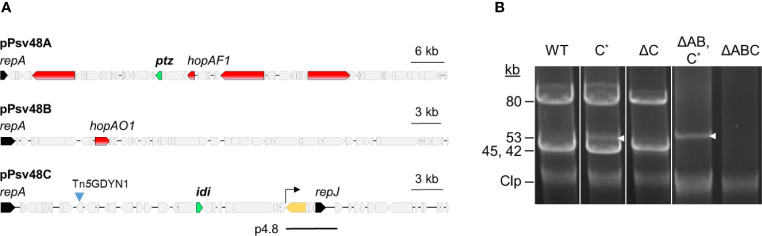
Plasmid profiles of plasmid-cured derivatives of *Pseudomonas* *syringae* pv. savastanoi NCPPB 3335. **(A)** Map of the three virulence plasmids of *Ps* pv. savastanoi NCPPB 3335, showing in red, two type III effector genes (*hopAF1* and *hopAO1*) and three putative virulence genes; in green, genes coding for cytokinin biosynthesis enzymes; in black, genes for replication initiator proteins; and in yellow, the only copy of IS*801* in pPsv48C, with the arrow indicating the direction of one-ended transpositions. The black line labeled as p4.8 indicates the position and extent of the deleted derivative of pPsv48C present in strain ΔC. **(B)** Uncut plasmids were separated by electrophoresis in agarose gels from the strains shown. Strains: WT, wild type NCPPB 3335; letters after the Δ symbol indicate the plasmid(s) missing in each strain; C*, pPsv48C::Tn*5*-GDYN1, indicated by white arrowheads. Clp, chromosome and linearized plasmids.

Strain NCPPB 3335 contains three native plasmids (A, B, and C) carrying several genes of relevance for the interaction with the plant host (**Figure 3A**) (Bardaji et al., 2017; Castañeda-Ojeda et al., 2017a; Castañeda-Ojeda et al., 2017b). We therefore wanted to construct a derivative of NCPPB 3335 cured of the three native plasmids to analyze their possible collective contribution to virulence and as a tool for future genetic analyses. For clarity, from now on we will designate bacterial strains with the letter Δ and the plasmid(s) or gene(s) that they are missing, with their collection names shown in **Supplementary Table S1**.

We constructed a derivative of strain ΔAB containing plasmid C tagged with Tn*5-*GDYN1 (**Figure 3**), which carries a *sacB* gene conferring lethality in the presence of 5% sucrose. To facilitate loss of plasmid C, its toxin–antitoxin systems were functionally neutralized by overexpressing the corresponding antitoxins from plasmid pRK3C (Tc^R^) (**Supplementary Table S1**).

The examination of the plasmid profile of more than 700 sucrose resistant (suc^R^) clones revealed that all lacked the band corresponding to plasmid C and contained a smaller plasmid of 4–15 kb. PCR, and DNA hybridization showed that these small plasmids were all deletion derivatives of pPsv48C, originating by anomalous, one-ended transposition events of IS*801* (data not shown). Several suc^R^ clones were propagated six times serially on LB without selection. A colony out of >300 tested was Tc^s^, and its plasmid profile indicated the loss of both pRK3C and the pPsv48C deleted derivative (**Figure 3B**); this clone was retained and designated ΔABC. The genome sequence of ΔABC confirmed that it was free of the three plasmids and contained no apparent reorganizations or major indels, as assessed by a genome comparison with strain NCPPB 3335 using WebACT (data not shown).

The same procedure as above was followed to obtain a derivative of NCPPB 3335, cured of only pPsv48C::Tn*5*-GDYN1. We were unable to entirely cure this plasmid but generated a strain containing a 4.8 kb plasmid (coordinates 27019–31852 of FR820587), resulting from a major deletion event of pPsv48C and lacking pRK3C (plasmid p4.8; **Figure 3**). We will refer to this strain as ΔC because this 4.8 kb deletion plasmid only contains an IS*801*, a hypothetical protein and replicon RepJ, carrying no known or putative virulence genes.

In summary, we were able to evict a highly stable plasmid tagged with a counterselection marker (gene *sacB*), by selection for survival in sucrose combined with functional inactivation of toxin–antitoxin systems. Given the relevance of native plasmids in plant pathogenic bacteria, the high stability provided by toxin–antitoxin systems (Bardaji et al., 2019) and their widespread presence in native plasmids (Vivian et al., 2001; Sundin, 2007; Shidore and Triplett, 2017; Bardaji et al., 2019), our methodology shall be of general applicability for plasmid curing for genetic and functional studies.

### Plasmid pPsv48C From NCPPB 3335 Is Essential for Tumor Formation in Olive

Inoculation of plasmid-cured strains allows for a rapid evaluation of the joint contribution of a large number of genes to virulence. In strain NCPPB 3335, plasmids A, B, and C contain diverse putative virulence genes, including two type III secretion system effector (T3E) genes and genes *ptz* (plasmid A) and *idi* (plasmid C) (**Figure 3A**; Bardaji et al., 2011). Our previous results indicated that plasmid A was necessary for tumor formation in olive (Bardaji et al., 2011), and we evaluated here the potential individual contribution of the other two plasmids.

**Figure 4:**
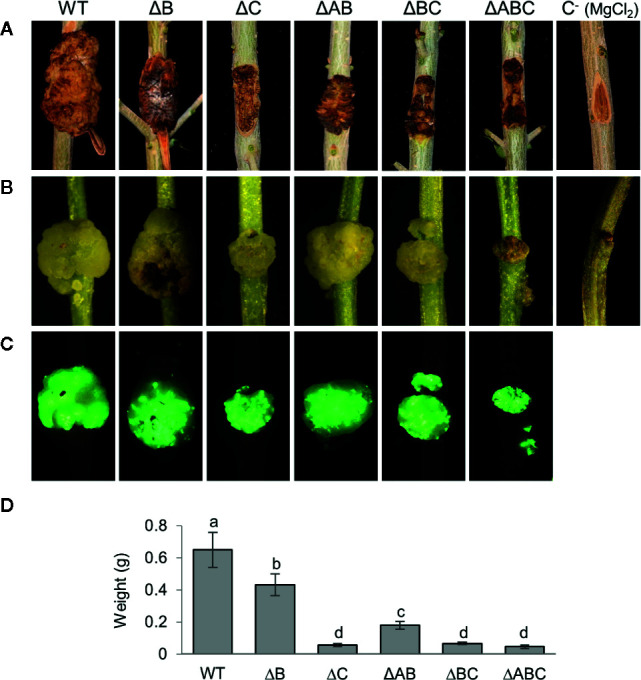
Symptoms induced in olive plants by NCPPB 3335 derivatives strains cured of native plasmids. **(A)** Knots developed in one-year-old lignified olive plantlets 90 days post-inoculation (dpi) by the indicated strains. **(B)** Knots developed in micropropagated olive plantlets by the indicated GFP-tagged strains at 28 dpi and **(C)** complementary epifluorescence microscopy images of knots. **(D)** Quantification of knot weight generated by the indicated strain. Error bars represent the standard deviation from the average weight of a minimum of three knots. The same letters indicate differences that were not significant (α = 0.05), using a two-way ANOVA followed by the Duncan’s test. Experiments were carried out in triplicate, with three inoculated plants per strain and experiment. Strains: WT, NCPPB 3335; letters after the Δ symbol indicate the plasmid(s) missing in each strain; C−, negative control inoculated with MgCl_2_.

All strains developed similar symptoms in inoculated one-year-old and micropropagated olive plants (**Figure 4**). As described, the wild-type strain NCPPB 3335 induced typical tumors, whereas strain ΔAB induced severely reduced tumors (Rodríguez-Moreno et al., 2009; Bardaji et al., 2011; Castañeda-Ojeda et al., 2017b). Strain ΔB induced tumors of a size and weight between those produced by the wild-type strain (strain NCPPB 3335) and ΔAB. Additionally, tumors produced by this strain in micropropagated plants were of wild-type size but slightly necrotic. These phenotypes agree with previous results, demonstrating that loss of the T3E gene *hopAO1* located in plasmid B leads to smaller and necrotic tumors (Castañeda-Ojeda et al., 2017b), and suggest that plasmid B does not contain other relevant virulence genes. Strains ΔC and ΔBC both lack plasmid C and produced tumors with a further significant weight reduction over the previous strains. Strain ΔABC, lacking the three plasmids, produced symptoms that were indistinguishable from those produced by strains lacking plasmid C.

Additionally, all the strains used in inoculation experiments were tagged with the green-fluorescent protein (GFP) using plasmid pLRM1-GFP (Rodríguez-Moreno et al., 2009). This suggests that all wild type and mutant strains are able to efficiently colonize the tumors (**Figure 4**). The patchy fluorescence pattern of some of the plasmid-cured strains is difficult to interpret and could be due to a restricted distribution of bacterial populations or to the already described dynamic evolution of fluorescence (Rodríguez-Moreno et al., 2009).

In summary, these results indicate that strain NCPPB 3335 contains three functional virulence plasmids that are clearly essential for the elicitation of full symptoms in its plant host.

### Genes *ptz* and *idi* Are Essential to Produce Tumors in Olive Plants

Since manipulation of the levels of phytohormones is central in the interaction with plants of diverse phytopathogens (Ma and Ma, 2016), we hypothesized that the phenotypes displayed by strains devoid of plasmids A and/or C could be due, at least in part, to the lack of genes *ptz* (plasmid A) and *idi* (plasmid C), which are likely involved in the biosynthesis of CKs. To address this hypothesis, we constructed single-knockout *ptz* (Δptz) and *idi* (Δidi) mutants, and a double-knockout mutant *ptz* *idi* (Δptz-idi) (**Supplementary Table S1**). For complementation analyses, we constructed three vectors containing genes *ptz* and *idi* cloned individually or together, and expressed from their own promoters. In the plasmid containing only gene *ptz*, this gene was also expressed from the constitutive P*_lac_* promoter of the vector. Gene *idi* is the second CDS of a possible operon including a putative methyltransferase gene (PSPSV_C0023); we therefore constructed complementation plasmids containing only gene *idi* or both, PSPSV_C0023 and *idi*. In the first case, gene *idi* was cloned plus a 578 nt fragment upstream of its start codon that contains two putative promoters, as predicted by the BPROM software (**Supplementary Figure S1**); importantly, the fragment was cloned behind a strong transcriptional terminator, ensuring that transcription must originate from within the insert.

**Figure 5:**
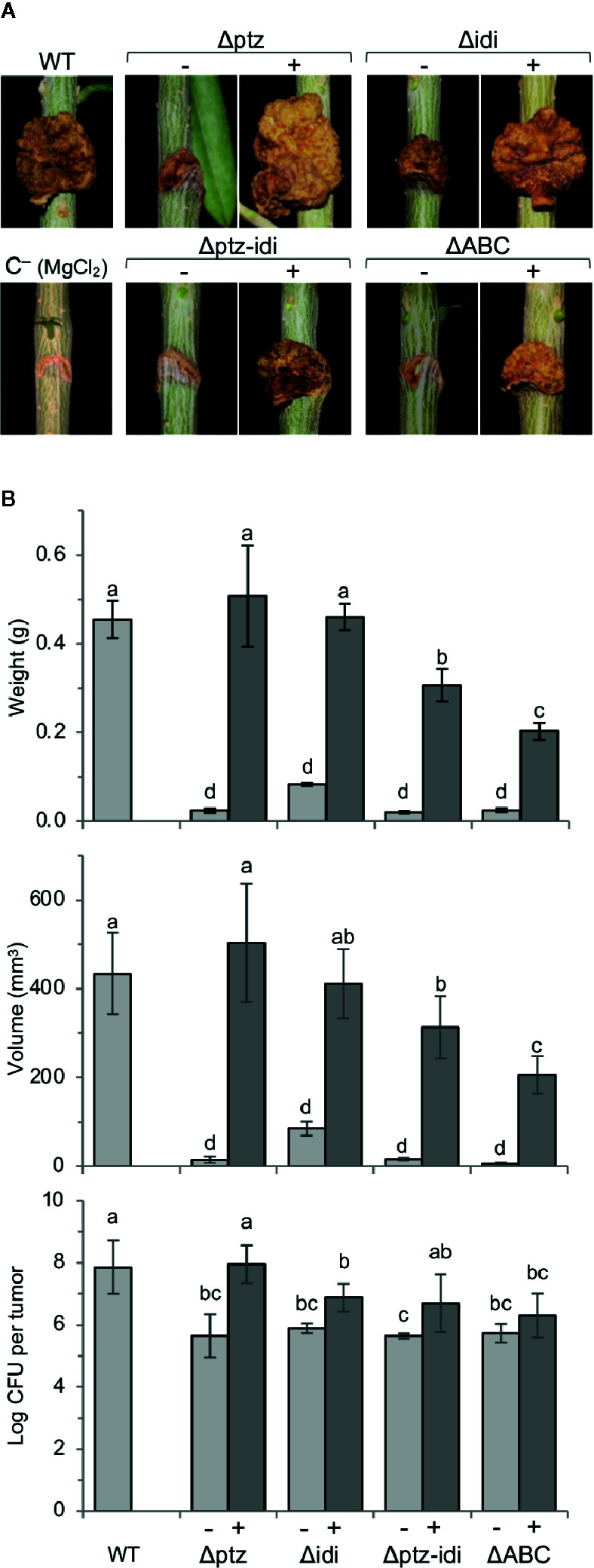
Genes *ptz* and *idi*, coding for enzymes for cytokinin biosynthesis, are essential for the induction of symptoms in one-year-old olive plants. **(A)**, Symptoms induced in lignified olive plantlets 90 days after inoculation. **(B)**, Average weight, volume, and bacterial population of at least three tumors induced by the indicated strains, with bars indicating standard deviation; different letters above bars indicate means that are significantly different according to a two-way ANOVA (P ≤ 0.05) followed by the Duncan’s test. WT, strain NCPPB 3335. Symbols (−) and (+) indicate without or with complementation, respectively, with the deleted gene(s) cloned in a broad host-range vector. Results represent the mean of at least two experiments, each with three olive plants inoculated per strain.

We evaluated the contribution of genes *ptz* and *idi* for virulence in both lignified one year-old olive plants (**Figure 5**) and micropropagated olive plants (**Supplementary Figure S5**), the latter routinely used for virulence assays because they show a higher susceptibility to olive knot disease (Rodríguez-Moreno et al., 2008). Plants inoculated with the wild-type strain NCPPB 3335 developed typical tumors in both plant systems, which were of 434 ± 91 and 24 ± 2 mm^3^ average volume in lignified and micropropagated plants, respectively, similar to previous results (Aragón et al., 2014). In contrast, strain ΔABC and mutants Δptz, Δidi and Δptz-idi induced barely visible tumors that were significantly smaller than those elicited by the wild-type strain, with mean volumes of 6–85 and 7–11 mm^3^ in lignified (**Figure 5**) and micropropagated plants (**Supplementary Figure S5**), respectively.

In micropropagated olive plants, complementation of tumor volume was only partial for all mutants (**Supplementary Figure S5**). However, in lignified plants the cloned genes *ptz* and *idi* completely reversed the phenotype of the individual mutants. Differences in complementation of mutants using both types of plants have already been observed for other virulence factors (Aragón et al., 2015a; Aragón et al., 2015b; Caballo-Ponce et al., 2017b) and could be due to a different hormonal regulation between micropropagated and lignified plants. Although the low virulence phenotype of strain Δidi was also fully complemented by the vector containing the fragment with genes PSPSV_C0023 and *idi* (**Supplementary Figure S1** and data not shown), the observed complementation with gene *idi* cloned by itself indicates that this gene is immediately preceded by a functional promoter and likely not included in an operon. In lignified plants, complementation of the double mutant Δptz-idi and the plasmidless strain ΔABC with the plasmid encoding both *ptz* and *idi* was also only partial (**Figure 5**). This could be due to a lower level of expression of *ptz* when it was cloned with gene *idi* than when it was cloned singly, because in this last case it was also transcribed from the constitutive P*_lac_* promoter from the vector. Additionally, the lower virulence of the complemented strain ΔABC could be due to the lack of other plasmid-borne genes that might contribute to the tumorigenesis. In fact, tumors developed in micropropagated plants infected with strain ΔABC or its complemented derivative were necrotic (**Supplementary Figure S5**), likely because they lack the T3E gene *hopAO1* (Castañeda-Ojeda et al., 2017b).

In both plants systems, total bacterial populations in tumors were significantly higher for the wild-type strain than for strain ΔABC or for the Δptz and Δidi mutants (**Figure 5B**, **Supplementary Figure S5**). In most cases, total bacterial counts were fully recovered by complementation, with the exception of the double Δptz-idi mutant in micropropagated olive plants (**Supplementary Figure S5**) and strain ΔABC and the Δidi mutant in lignified plants (**Figure 5**), which were recovered only partially. The population densities of the strains were also calculated per gram of plant tissue, in order to analyse whether the mutant strains multiplied successfully in olive plants but failed to produce knots. However, the number of cfu/g of plant tissue reached by all mutant strains was approximately one order of magnitude lower than that of the wild-type strain (**Supplementary Figure S6**), further demonstrating that lack of *ptz* and *idi* affects the ability of the strains to multiply and/or survive in olive tissues.

Together, these results unequivocally show that both *ptz* and *idi* are individually essential for the development of tumors in olive by Psv NCPPB 3335. Nevertheless, their contribution to virulence might be at least partially additive because the double mutant produced somewhat smaller tumors than either single mutant.

### Gene *ptz* Is Essential for the Biosynthesis of Cytokinins, but Not Gene *idi*

**Figure 6:**
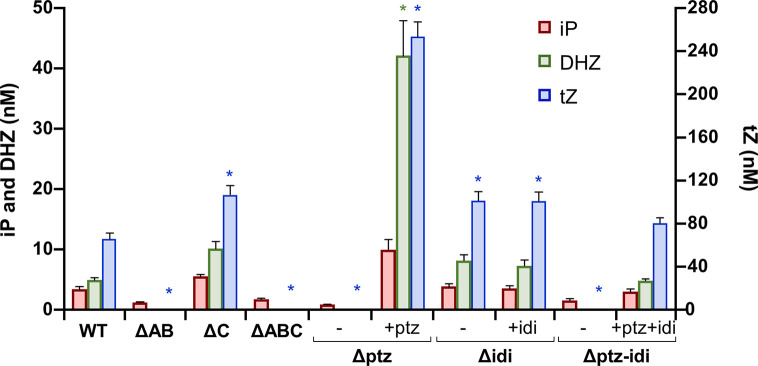
Cytokinins produced in minimal medium by *Pseudomonas syringae* pv. savastanoi NCPPB 3335 and derivative mutants. WT, strain NCPPB 3335; letter Δ indicates the plasmid(s) or gene(s) missing in each mutant strain derived from NCPPB 3335; symbols (−) and (+) indicate without or with complementation, respectively, with genes *ptz* and/or *idi* cloned in a broad host-range vector. Bars indicate nM concentrations of each cytokinin, with *trans*-zeatin (tZ) values to the right, and isopentenyladenine (iP) and dihydrozeatin (DHZ) values to the left. Results represent the average of three separate cultures, with two replicas each, and the standard error. Asterisks indicate significant differences from WT within each type of cytokinin (α = 0.05), using a two-way ANOVA followed by the Duncan’s test.

Cultures filtrate from the wild-type strain NCPPB 3335 contained iP, iPR, DHZ, DHZR, tZ and tZR, as well as their monophosphate precursors and glycosylated forms of tZ and DHZ (**Figure 6**, **Supplementary Table S3**). From these, the most active form tZ and its riboside tZR were the most abundant CKs. Likewise, and adding to the variability in the CKs spectrum produced by different Psv strains, we also detected minute amounts of *cis*-zeatin (cZ) and substituted forms in culture filtrates from strain NCPPB 3335 (**Supplementary Table S3)**.

Culture filtrates from strains lacking plasmid A or those with the inactivated *ptz* gene did not produce tZ or DHZ or their precursors, but still produced significant amounts of iP (**Figure 6**, **Supplementary Table S3**). In agreement with this, mutants of Psv lacking plasmids carrying gene *ptz* have been previously described to still synthesize trace amounts of CKs and/or similar amounts of iP to their corresponding wild-type strain (MacDonald et al., 1986; Iacobellis et al., 1994). Conversely, strains lacking gene *idi* produced significantly higher amounts of tZ and similar amounts of the other CKs to the wild-type strain. Additionally, all mutants produced similar amounts of cZ CKs to strain NCPPB 3335. Complementation of site-directed mutants restored the ability to synthesize CKs (**Figure 6**), although to higher levels than in the wild-type strain. This is probably due to the genes being cloned in a plasmid, leading to a higher gene copy number and a likely higher gene expression. Additionally, expression of gene *ptz* cloned by itself is also driven by the constitutive *P_lac_* promoter from the vector. Nevertheless, tZ was in all cases still the most abundant CK in culture filtrates. Finally, the patterns of CKs found in filtrates from strains ΔC and Δidi were essentially identical, as well as with the pairs ΔAB/Δptz and ΔABC/Δptz-idi. This suggests that these plasmids do not contain other genes that might be involved in the biosynthesis of CKs.

Together, these results indicate that gene *ptz*, but not gene *idi*, is essential for the biosynthesis of high amounts of *trans*-zeatin, isopentenyladenine and dihydrozeatin CKs and their substituted forms. In turn, the activity of gene *idi* influences the relative amounts of tZ in the CK cell pool.

## Discussion

Genes *ptz* and *idi* code for enzymes of the CK biosynthesis pathway, and here we show that they are both essential for the induction of full symptoms in olive plants by *P. syringae* pv. savastanoi NCPPB 3335 and for reaching high *in planta* bacterial populations. The use of plasmid-cured strains also showed that, although containing genes contributing to virulence (Bardaji et al., 2011; Castañeda-Ojeda et al., 2017a; Castañeda-Ojeda et al., 2017b), its three native plasmids do not contain any other gene(s) that are essential for CKs biosynthesis and tumorigenesis. Additionally, the wild-type strain NCPPB 3335 and its *ptz*, *idi*, and *ptz*–*idi* mutants produced similar amounts of IAA under the same conditions used to quantify CKs (data not shown), suggesting that the observed phenotypes are not due to changes in auxin production. The scientific literature has largely assumed that the production of CKs contributes to virulence by Psv, mostly because the occurrence of large deletions involving gene *ptz* or curing of plasmids containing this gene lead to a reduced virulence (MacDonald et al., 1986; Iacobellis et al., 1994; Bardaji et al., 2011). However, native plasmids from the *P. syringae* complex usually carry a large diversity of genes required for pathogenicity or full virulence, such as T3E genes (Jackson et al., 1999; Vivian et al., 2001; Sundin, 2007; Bardaji et al., 2011). Therefore, direct proof of the involvement of gene *ptz* in, or the requirement of CKs for, virulence was lacking.

As previously reported for other Psv strains (Surico et al., 1985b; MacDonald et al., 1986; Powell and Morris, 1986; Iacobellis et al., 1994; Cinelli et al., 2014), NCPPB 3335 produces diverse CKs *in vitro*, of which tZ was by far the most abundant. Unlike previous reports with other strains, we were also able to detect cZ and substituted forms in cultures of NCPPB 3335; it is not known if this is a peculiarity of this strain or if it could be a more general feature. Free-base natural CKs, such as tZ, iP, and DHZ, have been shown to exhibit biological activity and are regarded as the active forms, whereas other modified forms of these CKs as well as cZ are considered to have at most a low level of activity (Sakakibara, 2010). Using site-directed mutagenesis, we demonstrate that gene *ptz* is essential for the biosynthesis of high amounts of tZ, iP, and DHZ in culture; thus, it is feasible that *trans*-zeatin and/or its modified forms are involved in tumorigenesis *in planta*. Likewise, the tZ synthesizing gene from *Agrobacterium tumefaciens*, its CK products, or both, participate in the regulation of diverse virulence factors and are also needed for full tumorigenesis potential and *in planta* bacterial growth (Hwang et al., 2013).

Mutants lacking gene *idi* were still able to synthesize the same spectrum of CKs as the wild type (**Figure 6**, **Supplementary Table S2**), which agrees with previous results, showing that expression of *ptz* from Psv in *E. coli* was sufficient to produce at least certain CKs (MacDonald et al., 1986; Powell and Morris, 1986). However, the *idi* mutant and the strain lacking plasmid C (ΔC) produce significantly higher amounts of tZ than the wild-type strain; it is possible that this could be due to alterations in the concentration of the CKs precursors DMAPP and/or HMBDP, originating from the lack of the *idi* gene (**Figure 1**). Nevertheless, Ptz from NCPPB 3335 is closely related to the two isopentenyl transferases of *Agrobacterium* *tumefaciens*, coded by genes *tmr* and *tzs* (Powell and Morris, 1986), and Tmr uses HMBDP preferentially as a substrate (Sakakibara, 2006). Therefore, it is likely that Ptz also preferentially uses HMBDP. If this were the case, a mutation in *idi* would not necessarily impact the generation of adequate substrates for Ptz (**Figure 1**). The higher amount of tZ found in the *idi* mutants could then suggest that, under natural conditions, the activity of gene *idi* and/or its product leads to a reduction in the biosynthesis of tZ and DHZ in the wild-type strain NCPPB 3335, perhaps by altering the amount of substrates available in the cell. Independently of the mechanism involved, it is likely that *idi* might contribute to ensure a correct balance of the different CKs *in planta*, or to promote the synthesis of adequate substituted forms, to promote tumorigenesis. Thus, it could be possible that the inability of strains Δidi and ΔC to induce tumors in olive plants (**Figure 5**, **Supplementary Figure S5**) is a consequence of their altered levels of tZ, compared to those of the wild-type strain (**Figure 6**). Additionally, it would be worth investigating possible changes in the olive immune response towards the *ptz* and *idi* mutants because it is well-established that CKs regulate plant defense responses in a dosage-dependent manner (Ma and Ma, 2016; Spallek et al., 2018).

The lack of genes *ptz* and/or *idi* also affected the ability of the strains to grow and survive in olive tissues (**Figure 5**, **Supplementary Figure S5**), suggesting that reduction of the total bacterial population in the host might also affects tumor size. Other authors have reported that *P. syringae* pv. nerii strains cured of plasmids encoding *ptz* are able to multiply at a rate similar to the wild-type strain in oleander leaves (Iacobellis et al., 1994). Furthermore, *P. savastanoi* strains isolated from *Myrtus* lacking gene *ptz* and producing undetectable amounts of CKs successfully invaded the host tissues and moved systemically, apparently unaffected by host defenses, such as the synthesis of tannins and the lignin deposits around the inoculation sites (Schiff et al., 2019). Thus, the role of CKs in the ability of *P. savastanoi* to multiply and survive in plant tissues might be strain- and/or host-dependent and could be influenced by other virulence factors varying among strains (Moreno-Pérez et al., 2020). An attractive possibility is that genes involved in CKs biosynthesis in Psv, including *idi*, might be regulating the expression of other virulence genes, as has been shown for certain Psv virulence genes upon exogenous IAA treatment (Aragón et al., 2014).

Indoleacetic acid is synthesized by strains of the *P. syringae* complex through three different metabolic pathways and by different, unrelated sets of genes (Howden et al., 2009; Aragón et al., 2014; McClerklin et al., 2018). The biosynthesis of CKs by Psv appears to also be complex and depend on several genes. First, the cloned *ptz* gene from a Psv strain allowed *E. coli* to synthesize tZ, iP and iPR, but not ribosyl-1”-methylzeatin, which was synthesized by the parental Psv strain (MacDonald et al., 1986; Powell and Morris, 1986). Therefore, it is possible that an additional gene(s) different from *ptz* is responsible in Psv strains for the production of methyl CKs derivatives. Incidentally, a differential distribution of this gene in the pathogen population could explain why certain Psv strains synthesize the methylated forms whereas other strains do not (MacDonald et al., 1986; Cinelli et al., 2014). It is tempting to speculate that the methyltransferase gene found adjacent to gene *idi* could participate in the methylation of CKs found in Psv (Surico et al., 1985a; Evidente et al., 1986; MacDonald et al., 1986), although it would be necessary to support this with adequate experimental evidence. Secondly, although gene *ptz* is essential for the production of high amounts of CKs by strain NCPPB 3335 in culture, the Δptz mutants still synthesized significant amounts of iP and traces of other CKs (**Supplementary Table S2**). This has also been reported for other Psv strains (MacDonald et al., 1986; Iacobellis et al., 1994), indicating that Psv must contain other gene(s) involved in the biosynthesis of CKs, although in such a concentration that likely does not allow them to induce tumors. This scenario would be similar to the biosynthesis of IAA by strains of the *P. syringae* complex. A large proportion of *P*. *syringae* strains produce small amounts of IAA that, at least in the case of *Ps* pv. tomato DC3000, contribute modestly to virulence, probably by helping to suppress plant defense responses (Glickmann et al., 1998; Ma and Ma, 2016; McClerklin et al., 2018). Additionally, some strains contain the *iaaMH* operon and are able to produce high amounts of IAA, which in certain cases allow the bacterium to induce tumors in the plant host (Glickmann et al., 1998; Aragón et al., 2014). It is thus conceivable that the ability to synthesize low amounts of CKs might be common in the *P. syringae* complex, perhaps participating in the suppression of plant defenses (Ma and Ma, 2016; Spallek et al., 2018), whereas only a few pathovars will have the ability to synthesize high amounts of CKs by means of gene *ptz* and to be tumorigenic. We have to stress that there are strains of diverse pathovars of the *P. syringae* complex that contain genes *iaaMH*, *ptz* and/or produce high amounts of IAA or CKs, but that do not induce tumors in their common plant hosts (Glickmann et al., 1998; Cinelli et al., 2014). These strains, therefore, might lack some of the numerous genes needed for tumorigenesis (such as genes for type III secretion system effectors, for Na^+^/Ca^2+^ exchange or for the metabolism of phenolics) or to not produce an adequate balance of phytohormones (Caballo-Ponce et al., 2017a; Moretti et al., 2019; Moreno-Pérez et al., 2020).

Genes *ptz* and *idi* have a limited distribution among bacteria (**Supplementary Figure S2**), and their possession is not correlated with the colonization of woody hosts or with the induction of tumors. In particular, we found homologs in the genomes of only a few bacteria of the *P. syringae* complex (**Figure 2**), although all infecting woody plants and belonging to PG3 (genomospecies 2) (Gardan et al., 1999; Gomila et al., 2017). Remarkably, no homologs, or only for one of the genes, were found in the genomes of certain pathovars that elicit tumors, such as *P. amygdali*, *P. meliae*, *P. syringae* pvs. dendropanacis or rhaphiolepidis (**Figure 2**). Additionally, we found both genes *ptz* and *idi* in the genomes of *P. syringae* pv. photiniae deposited in the NCBI, even though this pathovar produces leaf spots (Goto, 1983) and is not reported to induce tumors. These discrepancies might be due to one or more of these reasons: 1) that there is variability in the distribution of these genes among all the tumorigenic bacteria, being absent in a small proportion of strains, as occurs in pathovars nerii and savastanoi (Cinelli et al., 2014; Moreno-Pérez et al., 2020); in particular, the three genomes currently available for *P. amygdali* are for strain NCPPB 2607, which was shown to produce negligible amounts of CKs in culture (Iacobellis et al., 1990) and that might therefore naturally lack *ptz*; 2) that the genes remained in the non-assembled reads, which is possible given that these genes are often surrounded by repeated sequences and their exclusion from the assembly has already been reported for other draft genomes (Moreno-Pérez et al., 2020); 3) that the sequenced strain has been misidentified, which is somewhat common among sequenced strains of the *P. syringae* complex (Gomila et al., 2017) and likely has occurred with the *P. tremae* strain, which belongs to PG3 and should have clustered close to the other strains (Gardan et al., 1999; Gomila et al., 2017); 4) that CKs perform diverse functions contributing to bacterial fitness and not necessarily related to the elicitation of tumors (Spallek et al., 2018), perhaps explaining the presence of genes *ptz* and *idi* in many non-tumorigenic bacteria (**Supplementary Figure S2**); and 5) that the contribution of *ptz* and *idi* to tumor development could be fulfilled by the activity of other gene(s) in certain bacterial phytopathogens.

Genes *ptz* and *idi* have a complex evolutionary history within *P. syringae* PG3 (genomospecies 2) that involves *ptz* being acquired at least twice during evolution. Genes *ptz* and *idi* appear to have evolved separately for a long time, as shown by their disparate phylogenies, before being linked and incorporated in bacteria from PG3 (**Supplementary Figures S2** and **S4**). The tumorigenic bacteria of PG3 are all in a single phylogenetic clade of pathogens of woody hosts, and intermingled with other species and pathovars causing cankers or other types of diseases (Gomila et al., 2017; Dillon et al., 2019b). It is likely therefore, that the DNA fragment with the linked *ptz* and *idi* genes was acquired only once during evolution, but that one or both genes were lost numerous times. Evidence for the independent loss of *ptz* and/or *idi* comes from their patchy distribution among diverse pathovars, including *P. syringae* pvs. nerii and savastanoi (Iacobellis et al., 1998; Cinelli et al., 2014; Moreno-Pérez et al., 2020). The DNA fragment containing the linked *ptz* and *idi* is currently present in, at least, strains of *P. syringae* pv. myricae and carried by native plasmids (**Figure 2**, **Supplementary Figure S3**), which likely facilitated its acquisition. Afterwards, gene *ptz* was lost and, concurrently or independently, bacteria of the Psv clade acquired a different homolog of *ptz* (**Figure 2**). Definite proof that *ptz* and *idi* were ancestrally linked comes from the conservation of the last 27 nt of the *ptz* CDS in front of the gene preceding *idi* in Psv NCPPB 3335 (**Supplementary Figure S4**). Its acquisition by the Psv clade and by PG3 for a second time during evolution therefore supports the notion that gene *ptz* plays a significant role in the pathogenesis of *P. syringae* pvs. nerii, retacarpa, and savastanoi with their plant hosts.

In summary, this work firmly establishes the direct role of gene *ptz* and/or its products in the biosynthesis of CKs, as well as gene *idi*, for the induction of tumors on olive plants by Psv. We present evidence that genes *ptz* and *idi* were acquired, physically linked, exclusively by bacteria from PG3 and have likely been repeatedly lost. Upon loss of *ptz* during evolution, tumorigenic bacteria from the savastanoi clade acquired a divergent homolog of *ptz*. Our results also indicate the existence of alternative routes for the biosynthesis of iP and cZ that do not require genes *ptz* or *idi*.

## Data Availability Statement

The genome sequence generated in this work is available at the NCBI (https://www.ncbi.nlm.nih.gov/bioproject/638121) with the BioProject ID PRJNA638121 and the genome assembly accession ASM1391112v1.

## Author Contributions

CR and JM planned and designed the research and analyzed and interpreted the data. MA and AP performed the experiments. ND, LU, and ON provided all the cytokinin analyses. MA, AP, CR, and JM carried out bioinformatics analyses, designed and prepared Figures and Tables, and wrote the manuscript.

## Funding

AP, CR, and JM were supported by grants FPU14/05551, AGL2017-82492-C2-1-R and AGL2017-82492-C2-2-R, respectively, from *Ministerio de Ciencia, Innovación y Universidades* (Spain), cofinanced by the *Fondo Europeo de Desarrollo Regional* (FEDER). ND, LU, and ON were supported by grant CZ.02.1.01/0.0/0.0/16_019/0000827, within the program Research, Development and Education (OP RDE).

## Conflict of Interest

The authors declare that the research was conducted in the absence of any commercial or financial relationships that could be construed as a potential conflict of interest.

## Abbreviations

CKs, cytokinins; cZ, *cis*-zeatin; DHZ, dihydrozeatin; DHZR, dihydrozeatin riboside; DMAPP, dimethylallyldiphosphate; HMBDP, hydroxymethylbutenyl diphosphate; *idi*, isopentenyl-diphosphate delta-isomerase gene (locus_tag PSPSV_C0024); IAA, indoleacetic acid; iP, isopentenyladenine; iPR, isopentenyl adenosine; iPRMP, isopentenyladenosine 5′-monophosphate; MEP, methylerythritol phosphate pathway; MVA, mevalonate pathway; *ptz*, isopentenyl transferase gene (locus_tag PSPSV_A0024); tZ, *trans*-zeatin; tZR, *trans*-zeatin riboside; ZRMP, *trans*-zeatin riboside 5′-monophosphate.
