# Supplementary material for: Genes ptz and idi, Coding for Cytokinin Biosynthesis Enzymes, Are Essential for Tumorigenesis and In Planta Growth by P. syringae pv. savastanoi NCPPB 3335
Source: Front Plant Sci. 2020 Aug 21;11:1294. doi: 10.3389/fpls.2020.01294 (PMC7472798; doi:10.3389/fpls.2020.01294)
Supplement: Supplementary file 1 [file DataSheet_1.pdf]

(A)

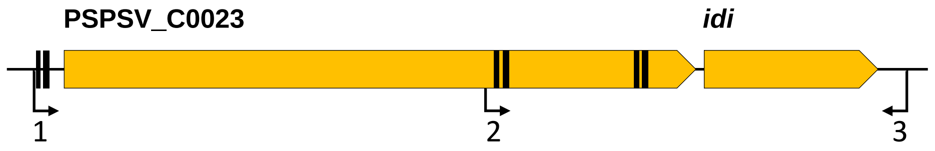

(B)

-35 -10

AGCGCTTGAGCTTTTGACTGAAAGCTCGATAAAACATTTTGTCTCTTTTATAGTCGGCTATCCTGGAGAA

AGCCCTGAGCTCTTCGAGGCGACCAGACGATTTCTTGTAAATGAGTTTTCTGGCGAATTTGCTTTGTATG

TTTGTATGTTGCAAGACGAGACTATGCCGCTTTGGGACGATGCGGAAAAGTTTAATATACAGGTTGATGA

CCCTAGCGGAGAGGCGCGTATCTGGAGCCACAGCGGCATGGACTCTAAAACTGCGAGGCAGTTGCAATTG

GAGACATTGCGTGAAGTTCGTTGGAACAATGACAACGCGATCTTCAGGTCATGGCAACATGATTTCGAGT

-35 -10

CTCCGCTGATTCCTGATAGGACACGGCAGGAGAACACCAGCCTGGAAAAACTAGTTGATCGACTTGGCAT

GATCAGCGTGGACATTAGCAGTGAGCAACAGGCGCGCAAACTTGAAGTCGAGATTACAAGCAAGCTGACT

###

GCGCATGGTATATCGGTTGTCCCTGATGCCGGTTTGAGCGGTTATATGCAAAAAGCTGAGAAATAATAAC

***

GAAAG<u>**GGAG**</u>TAGCCGGTA GTG AGT GAT GAG TAT GTC GTA TTA GTC GAC AAC GAC GAC

V S D E Y V V L V D N D D

Supplementary Figure S1. Organization of the *idi* gene from plasmid pPsv48C and location of putative promoters. **(A)** Genetic map showing the relative location of PSPSV_C0023 and the *idi* gene (PSPSV_C0024). Black arrows indicate the positions of primers iptSAM_F (1), iptC_F (2), and iptC_R (3), allowing the cloning of a DNA fragment with both genes or with only gene *idi* (see Table S2). Black boxes show the relative positions of -35 and -10 boxes of three relevant promoters predicted by the software BPROM. **(B)** Sequence from the start of primer iptC_F (2 in panel A). Boxes labelled as -35 and -10 correspond to the two rightmost promoters shown in panel A. Hashes indicate the stop codon for PSPSV_C0023, and asterisks indicate the predicted start codon of gene *idi*, with the corresponding translation below the triplets. A putative ribosome binding site for *idi* is in bold and underlined.
