# Supplementary material for: Genes ptz and idi, Coding for Cytokinin Biosynthesis Enzymes, Are Essential for Tumorigenesis and In Planta Growth by P. syringae pv. savastanoi NCPPB 3335
Source: Front Plant Sci. 2020 Aug 21;11:1294. doi: 10.3389/fpls.2020.01294 (PMC7472798; doi:10.3389/fpls.2020.01294)
Supplement: Supplementary file 2 [file DataSheet_2.pdf]

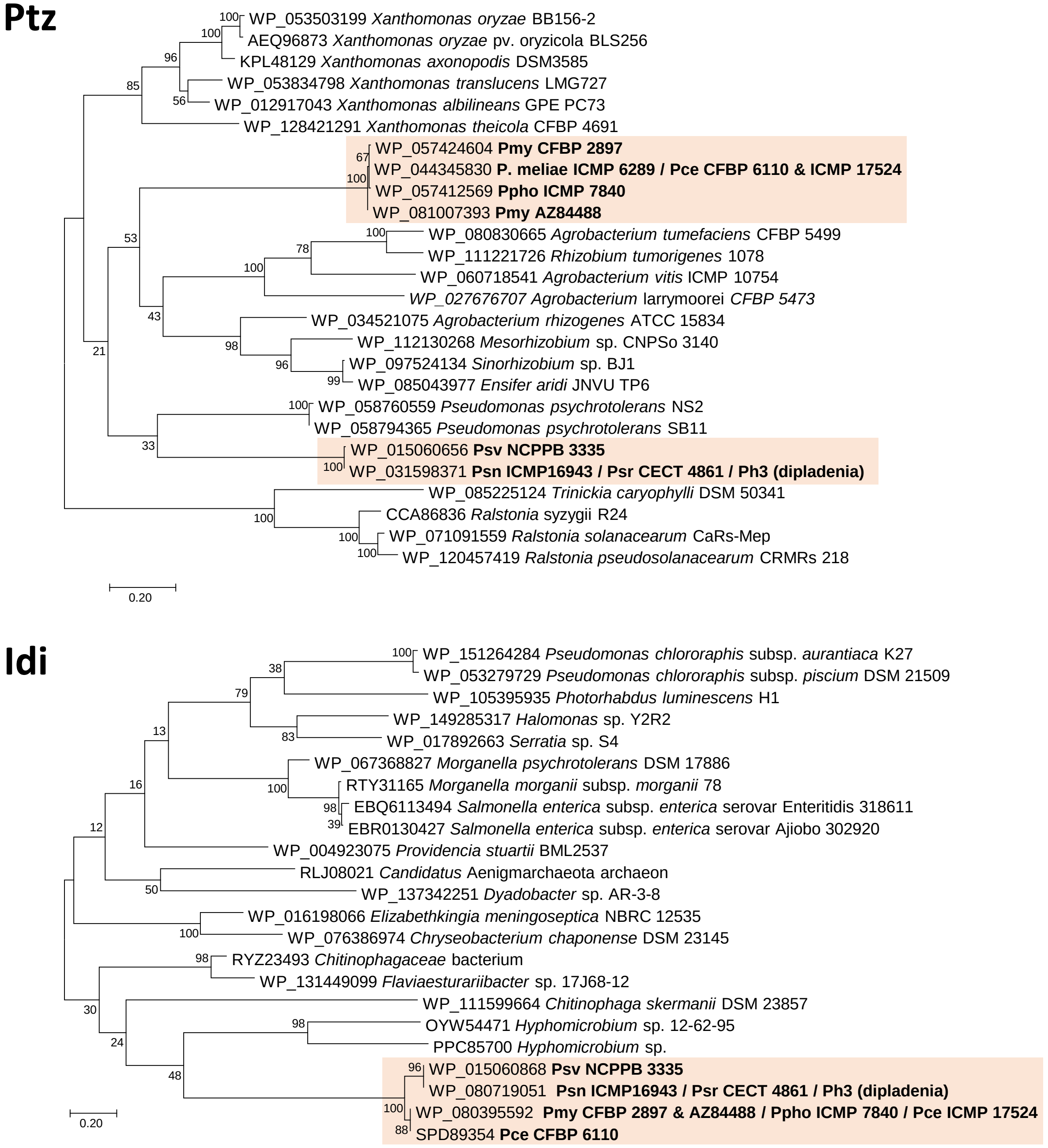

Supplementary Figure S2. Ptz and Idi evolved separately before invading *Pseudomonas syringae* PG 3 (genomospecies 2). Unrooted ML trees of *ptz* and *idi* gene products more closely related to those of *P. syringae* pv. savastanoi NCPPB 3335. The trees include examples from one strain from all bacterial species and pathovars containing homologs of these genes; species or pathovars containing both genes, within the genetic distances shown, were in all cases retained for tree reconstruction. Protein sequences are identified by their accession no. and example strains are indicated for identical protein groups. The trees were constructed in MEGA 7 using the JTT+G+I (Ptz) or the LG+G (Idi) models and using all sites; branch lengths are proportional to the number of substitutions per site, and numbers in nodes are bootstrap percentages of 200 replicates. In boldface and orange background, strains belonging to *Pseudomonas syringae* (Ps) genomospecies 2, with the following abbreviations for pathovars: Pce, cerasicola; Pmy, myricae; Psn, nerii; Ppho, photiniae; Psr, retacarpa; Psv, savastanoi. Ph3 (dipladenia), is a bacterial strain phylogenetically related to Psn and causing tumors in dipladenia (*Mandevilla* spp.) plants.
