# Supplementary material for: Genes ptz and idi, Coding for Cytokinin Biosynthesis Enzymes, Are Essential for Tumorigenesis and In Planta Growth by P. syringae pv. savastanoi NCPPB 3335
Source: Front Plant Sci. 2020 Aug 21;11:1294. doi: 10.3389/fpls.2020.01294 (PMC7472798; doi:10.3389/fpls.2020.01294)
Supplement: Supplementary file 3 [file DataSheet_3.pdf]

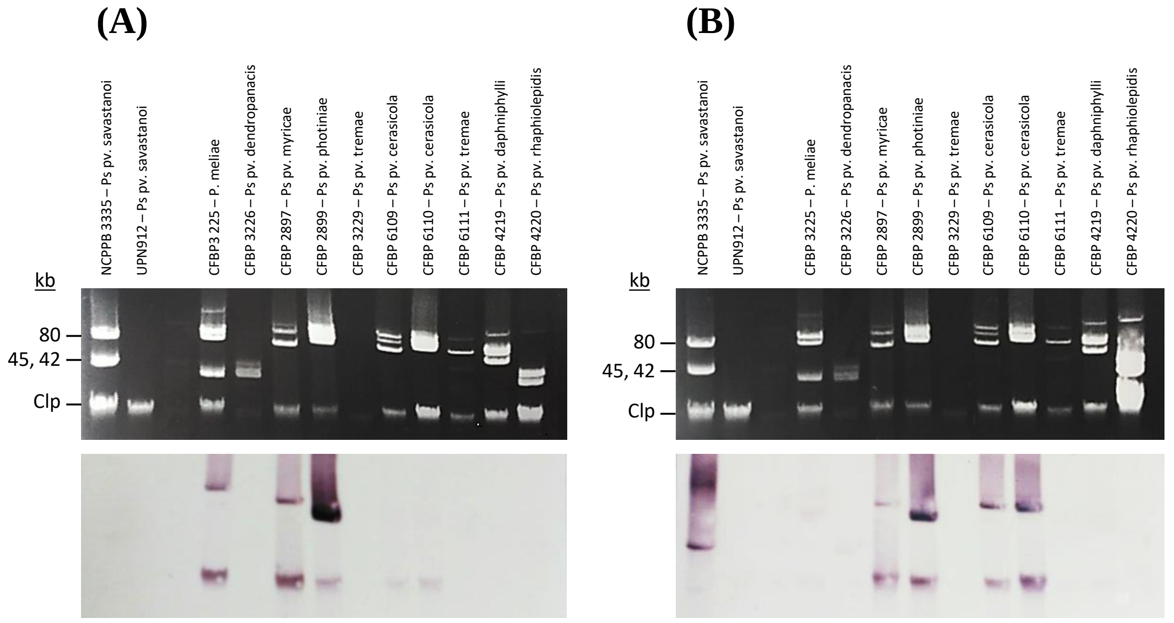

Supplementary Figure S3. Genes *ptz* and *idi* are carried by plasmids. Uncut native plasmids were separated by electrophoresis in 0.8 % agarose gels (upper panels), transferred to nylon membranes and subjected to Southern hybridization using internal probes for the *ptz* gene from *P. meliae* CFBP 3225 (**A**) and for the *idi* gene from *P. syringae* pv. savastanoi NCPPB 3335 (**B**). UPN912 (strain ΔABC) is a plasmidless derivative of strain NCPPB 3335. Clp, chromosome and linearized plasmids.
