# Supplementary material for: Genes ptz and idi, Coding for Cytokinin Biosynthesis Enzymes, Are Essential for Tumorigenesis and In Planta Growth by P. syringae pv. savastanoi NCPPB 3335
Source: Front Plant Sci. 2020 Aug 21;11:1294. doi: 10.3389/fpls.2020.01294 (PMC7472798; doi:10.3389/fpls.2020.01294)
Supplement: Supplementary file 4 [file DataSheet_4.pdf]

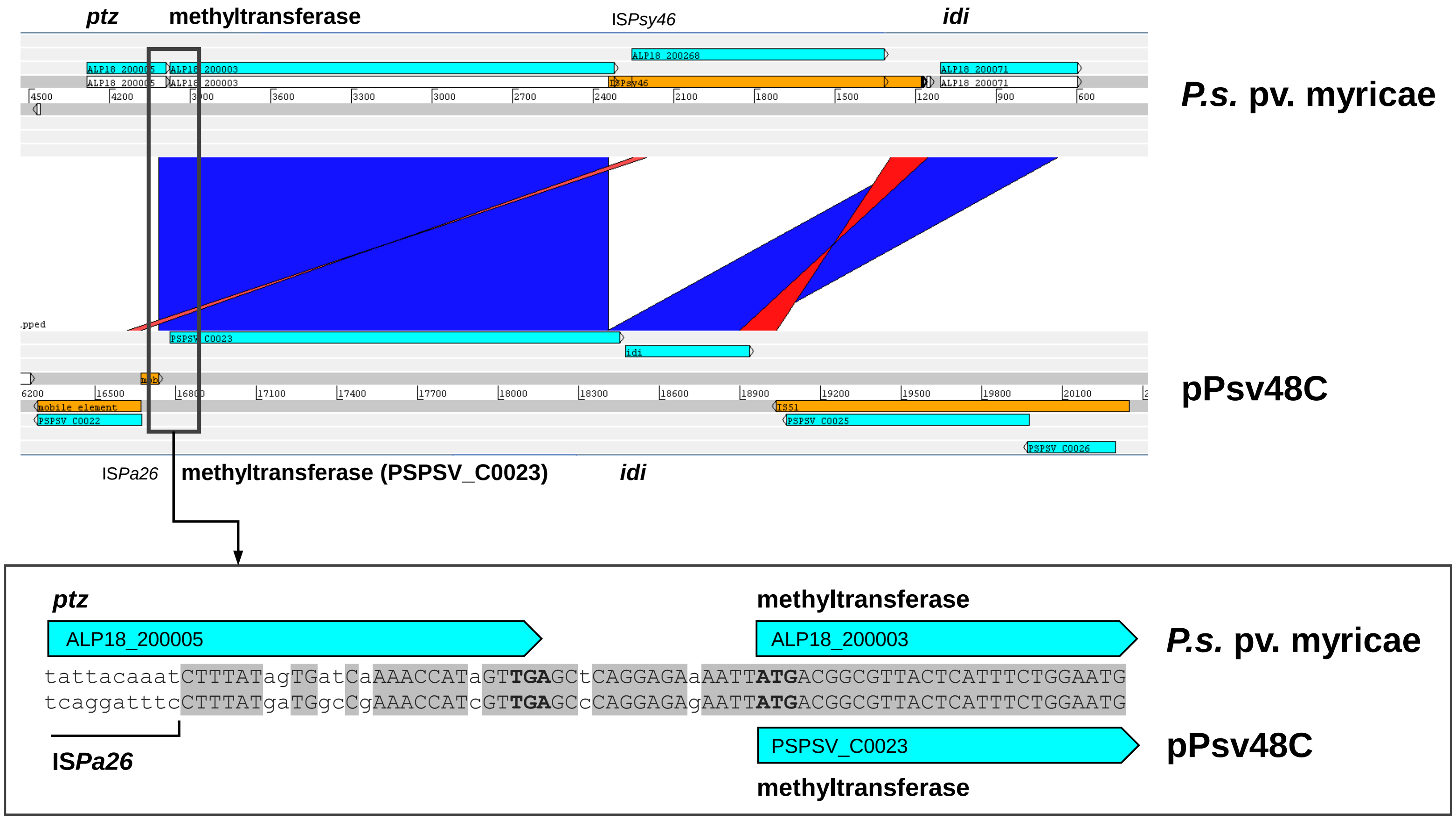

Supplementary Figure S4. Genes *ptz* and *idi* are ancestrally linked in *Pseudomonas syringae* genomospecies 2. Graphical representation (top) of a blastn comparison between a contig containing genes *ptz* (ALP18_200005) and *idi* (ALP18_200071) from *P. syringae* pv. myricae ICMP 11510 (accession no. RBUL01000272) and plasmid pPsv48C from *P. syringae* pv. savastanoi NCPPB 3335 (accession no. FR820587). Sequences were compared using blastn at the NCBI with default settings, except for a word size of 7, and relevant areas were visualized using ACT with blue and red shadings connecting inverted and direct homologous regions, respectively; only matches longer than 50 nt are shown. Relevant genes and elements are indicated above and below the sequences. Alignment (bottom) of the sequences overlapping the end of *ptz* and the start of the following CDS, approximately indicated at the top with a black rectangle. Identical bases in relevant regions are in capital letters with grey background. Stop and start codons are in boldface.
