# Supplementary material for: Genes ptz and idi, Coding for Cytokinin Biosynthesis Enzymes, Are Essential for Tumorigenesis and In Planta Growth by P. syringae pv. savastanoi NCPPB 3335
Source: Front Plant Sci. 2020 Aug 21;11:1294. doi: 10.3389/fpls.2020.01294 (PMC7472798; doi:10.3389/fpls.2020.01294)
Supplement: Supplementary file 5 [file DataSheet_5.pdf]

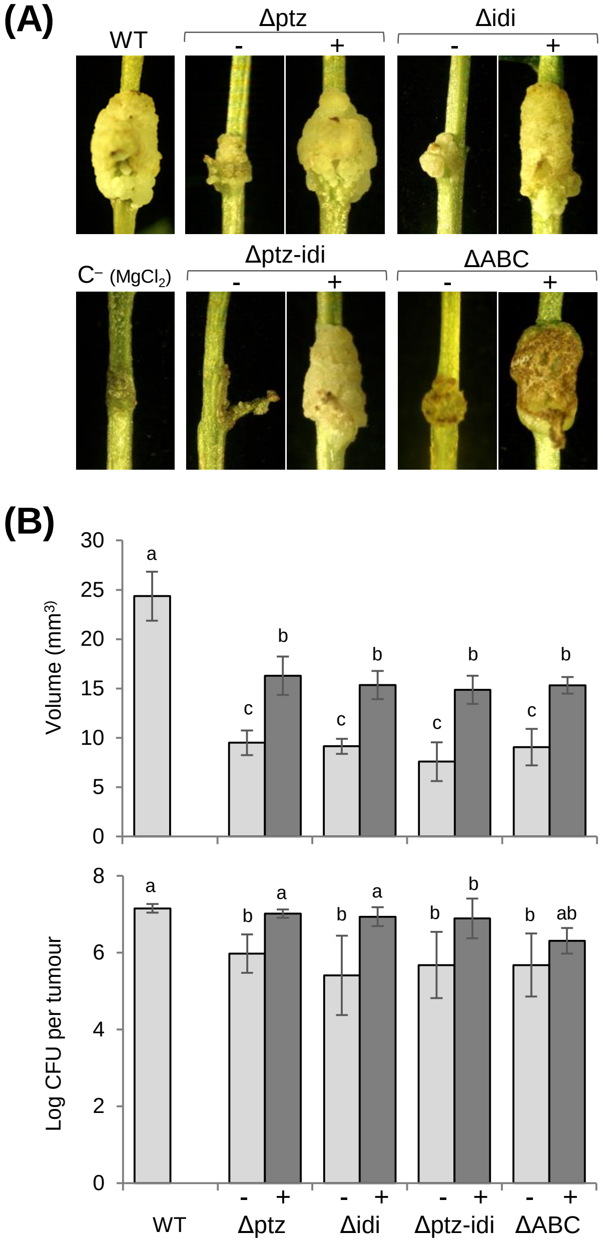

Supplementary Figure S5. Genes *ptz* and *idi* coding for enzymes for cytokinins biosynthesis, are essential for the induction of symptoms in micropropagated olive plantlets. **(A)** Symptoms induced in olive plantlets 90 days after inoculation. **(B)** Average volume and bacterial population of at least three tumors induced by the indicated strains, with bars indicating standard deviation; different letters above bars indicate means that are significantly different according to a two-way ANOVA ($P \leq 0.05$) followed by the Duncan's test. Strains: WT, NCPPB 3335; letters after the Δ symbol indicate the gene(s) or plasmids missing in each strain. Symbols (-) and (+) indicates without or with complementation with the deleted gene(s) cloned in a broad host-range vector, respectively. Results represent the mean of at least two experiments, each with three olive plants inoculated in two places per strain.
