# Supplementary material for: Genes ptz and idi, Coding for Cytokinin Biosynthesis Enzymes, Are Essential for Tumorigenesis and In Planta Growth by P. syringae pv. savastanoi NCPPB 3335
Source: Front Plant Sci. 2020 Aug 21;11:1294. doi: 10.3389/fpls.2020.01294 (PMC7472798; doi:10.3389/fpls.2020.01294)
Supplement: Supplementary file 6 [file DataSheet_6.pdf]

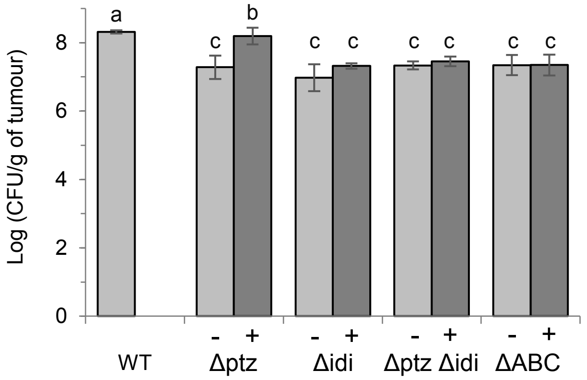

Supplementary Figure S6. Genes *ptz* and *idi*, coding for enzymes for cytokinins biosynthesis, affect the bacterial ability to proliferate in olive tumours in one-year-old olive plants. Average bacterial populations of at least three tumours induced by the indicated strains with respect to tumour fresh weight, with bars representing standard deviation; different letters above bars indicate means that are significantly different according to a two-way ANOVA ($P \leq 0.05$) followed by the Duncan's test. WT, strain NCPPB 3335. Symbols (-) and (+) indicates without or with complementation, respectively, with the deleted gene(s) cloned in a broad host-range vector. Results represent the mean of at least two experiments, each with three olive plants inoculated per strain.
