# Supplementary material for: Genes ptz and idi, Coding for Cytokinin Biosynthesis Enzymes, Are Essential for Tumorigenesis and In Planta Growth by P. syringae pv. savastanoi NCPPB 3335
Source: Front Plant Sci. 2020 Aug 21;11:1294. doi: 10.3389/fpls.2020.01294 (PMC7472798; doi:10.3389/fpls.2020.01294)
Supplement: Supplementary file 7 [file DataSheet_7.pdf]

Table S1. Strains and plasmids used in this work

Strain (abbreviation)[a]	Characteristics	Plasmids			Genes		Source or reference
		A	B	C	*ptz*	*idi*	
Escherichia coli							
NEB10β	Δ(*mrr-hsdRMS-mcrB*) *deoR recA1 endA1 araD139* Δ(*ara*, *leu*)7697 *galU galK* λ^{-} *rpsL nupG*						(Grant et al., 1990)
S17-1	Strain used to transfer pDR1 plasmid						(Flores et al., 1993)
***Pseudomonas syringae* pv. savastanoi**							
C* (UPN878)	NCPPB 3335 containing pPsv48C::Tn*5*-GDYN1-2	+	+	+	+	+	This work
NCPPB 3335	Pathotype strain, isolated from an olive (*Olea europaea*) tumor, France 1976, contains 3 native plasmids (pPsv48A, pPsv48B y pPsv48C); synonym Psv48	+	+	+	+	+	NCPPB[b]
ΔAB (Psv48ΔAB)	NCPPB 3335 cured of pPsv48A and pPsv48B	-	-	+	-	+	(Bardaji et al., 2011)
ΔAB, C* (UPN877)	Psv48ΔAB containing pPsv48C::Tn*5*-GDYN1-2	-	-	+	-	+	This work
ΔABC (UPN912)	Plasmidless, is Psv48ΔAB cured of pPsv48C	-	-	-	-	-	This work

ΔC (UPN954)	UPN878 with a very large deletion of pPsv48C, leaving only 4.8 kb of the plasmid (coordinates 27019-31852 in FR820587)	+	+	-[c]	+	-	This work
Δidi (UPN1022)	NCPPB 3335Δ*idi*	+	+	+	+	-	This work
Δidi +*idi* (UPN1024)	Δidi (UPN1022) containing p31idi	+	+	+	+	+	This work
Δptz (UPN1028)	NCPPB 3335Δ*ptz*	+	+	+	-	+	This work
Δptz +*ptz* (UPN1029)	Δptz (UPN1028) containing pIPMptz	+	+	+	+	+	This work
Δptz-idi (UPN1030)	Derived from Δptz (UPN1028) by transfer of pPsv48CΔidi from strain Δidi (UPN1022); double mutant Δ*ptz* Δ*idi*	+	+	+	-	-	This work
Δptz-idi +*ptz*+*idi* (UPN1032)	Δptz-idi (UPN1030) containing p31idiptz	+	+	+	+	+	This work
**Other *Pseudomonas***							
CFBP 2897	*Ps* pv. myricae	-	-	-	+	+	CFBP[d]
CFBP 2899	*Ps* pv. photiniae	-	-	-	+	+	CFBP[d]
CFBP 3225	*P. meliae*	-	-	-	+	-	CFBP[d2]
CFBP 3226	*Ps* pv. dendropanacis	-	-	-	-	-	CFBP[d]
CFBP 3229	*Ps* pv. tremae	-	-	-	-	-	CFBP[d]

CFBP 4219	*Ps* pv. daphniphylli	-	-	-	-	-	CFBP[d]
CFBP 4220	*Ps* pv. rhaphiolepidis	-	-	-	-	-	CFBP[d]
CFBP 6109	*Ps* pv. cerasicola	-	-	-	+	+	CFBP[d]
CFBP 6110	*Ps* pv. cerasicola	-	-	-	+	+	CFBP[d]
CFBP 6111	*Ps* pv. tremae	-	-	-	-	-	CFBP[d]
**Plasmids**							
p31idi	pME6031 containing gene *idi* (1117 nt fragment; coordinates 17895-19011); Tc^R				-	+	This work
p31idiptz	pME6031 containing genes *idi* (1117 nt fragment; coordinates 17895-19011) and *ptz* (1372 nt fragment; coordinates 28142-29513); Tc^R				+	+	This work
p41idiSAM	pME6041 containing genes *idi* and PSPSV_C0023; Km^R				-	+	This work
pBlueScript II SK	*E. coli* cloning vector; 2.96 kb, Amp^R						Stratagene
pDR1	Delivery vector for Tn5-GDYN1, based on pSUP2021; Km^R, Gm^R, Sm^R, Spc^R; confers sucrose-dependent lethality						(Flores et al., 1993)

pGEM-T Easy	*E. coli* cloning vector; 3 kb, Amp^R						Promega
pGEM-T-KmFRT-BamHI	Contains Km^R from pKD4; Ap^R Km^R						(Ortiz-Martín et al., 2010)
pHP45Ω	Source of cassette Spc^R, 2 kb						(Prentki and Krisch, 1984)
pIPMptz	pBBR1MCS-5 containing gene *ptz*; Gm^R				+	-	This work
pJET1.2	*E. coli* cloning vector 2.9 kb, Amp^R						Thermo Fisher Scientific
pLRM1-GFP	pBBR-MCS5::GFP; pBBR1-MCS5::$P_{A1/04/03}$-RBSII-GFPmut3*-T0-T1; Gm^R						(Rodríguez-Moreno et al., 2009)
pME6031	Broad host range cloning vector; 8.3 kb, Tc^R						(Heeb et al., 2000)
pME6041	Broad host range cloning vector; 5.6 kb, Km^R						(Heeb et al., 2000)
pPsv48A	Virulence plasmid of NCPPB 3335 (accession nº FR820585); with RepA-PFP replicon; 80.1 kb	+	-	-	+	-	(Bardaji et al., 2011)
pPsv48AΔptz	pPsv48A containing deleted gen *ptz* (positions 28,245 – 28,943 of FR820585); Km^R	+	-	-	-	-	This work
pPsv48B	Native plasmid of NCPPB 3335	-	+	-	-	-	(Bardaji et al., 2011)

	(accession no. FR820586); with RepA-PFP replicon; 45.2 kb						
pPsv48C	Virulence plasmid of NCPPB 3335 (accession no. FR820587); with RepA-PFP and RepJ replicons; 42.1 kb	-	-	+	-	+	(Bardaji et al., 2011)
pPsv48C::Tn*5*-GDYN1-2	pPsv48C containing Tn*5*-GDYN1 inserted at position 7,433; Km^R, Gm^R, Suc^S	-	-	+	-	+	This work
pPsv48CΔidi	pPsv48C containing deleted gen *idi* (positions 18,294 – 19,046 of FR820587); Spc^R	-	-	+	-	-	This work
pRK3C	pRK415 derivative containing genes PSPSV_C0050, PSPSV_C0008 and PSPSV_C0003 cloned in tandem in this order, each with their own promoter and under the control of the P_{lac} promoter from the vector; Tc^R						(Bardaji et al., 2019)
pRK415	Broad host range cloning vector; 10.5 kb, Tc^R						(Keen et al., 1988)

[a] Collection from the Laboratorio de Fitobacteriología, Universidad Pública de Navarra, UPN
[b] National collection of Plant Pathogenic bacteria, York , United Kingdom
[c] As indicated, the strain suffered a massive deletion of plasmid C, only retaining one of the replicons and adjacent DNA but no virulence genes; therefore, and since it does not contain gene *idi*, we considered this strain as free of plasmid C
[d] French Collection for Plant-associated Bacteria, France

Bardaji, L., Pérez-Martínez, I., Rodríguez-Moreno, L., Rodríguez-Palenzuela, P., Sundin, G.W., Ramos, C., et al. (2011). Sequence and role in virulence of the three plasmid complement of the model tumor-inducing bacterium *Pseudomonas savastanoi* pv. savastanoi NCPPB 3335. *PLoS ONE* 6(10), e25705. doi: 10.1371/journal.pone.0025705.

Bardaji, L., Añorga, M., Echeverría, M., Ramos, C., and Murillo, J. (2019). The toxic guardians — multiple toxin-antitoxin systems provide stability, avoid deletions and maintain virulence genes of *Pseudomonas syringae* virulence plasmids. *Mobile DNA* 10(1), 7. doi: 10.1186/s13100-019-0149-4.

Flores, M., Brom, S., Stepkowski, T., Girard, M.L., Dávila, G., Romero, D., et al. (1993). Gene amplification in *Rhizobium*: identification and *in vivo* cloning of discrete amplifiable DNA regions (amplicons) from *Rhizobium leguminosarum* biovar *phaseoli*. *Proc. Natl. Acad. Sci. USA* 90, 4932-4936. doi: 10.1073/pnas.90.11.4932.

Grant, S.G., Jessee, J., Bloom, F.R., and Hanahan, D. (1990). Differential plasmid rescue from transgenic mouse DNAs into *Escherichia coli* methylation-restriction mutants. *Proc. Natl. Acad. Sci. USA* 87(12), 4645-4649. doi: 10.1073/pnas.87.12.4645.

Heeb, S., Itoh, Y., Nishijyo, T., Schnider, U., Keel, C., Wade, J., et al. (2000). Small, stable shuttle vectors based on the minimal pVS1 replicon for use in gram-negative, plant-associated bacteria. *Mol. Plant-Microbe Interact.* 13(2), 232-237. doi: 10.1094/mpmi.2000.13.2.232.

Keen, N.T., Tamaki, S., Kobayashi, D., and Trollinger, D. (1988). Improved broad-host-range plasmids for DNA cloning in Gram-negative bacteria. *Gene* 70, 191-197. doi: 10.1016/0378-1119(88)90117-5.

Ortiz-Martín, I., Thwaites, R., Macho, A.P., Mansfield, J.W., and Beuzón, C.R. (2010). Positive regulation of the Hrp type III secretion system in *Pseudomonas syringae* pv. phaseolicola. *Mol. Plant-Microbe Interact.* 23, 665-681. doi: 10.1094/mpmi-23-5-0665.

Prentki, P., and Krisch, H.M. (1984). *In vitro* insertional mutagenesis with a selectable DNA fragment. *Gene* 29, 303-313. doi: 10.1016/0378-1119(84)90059-3.

Rodríguez-Moreno, L., Jiménez, A.J., and Ramos, C. (2009). Endopathogenic lifestyle of *Pseudomonas savastanoi* pv. savastanoi in olive knots. *Microbial Biotechnology* 2(4), 476-488. doi: 10.1111/j.1751-7915.2009.00101.x.
