# Supplementary material for: Genes ptz and idi, Coding for Cytokinin Biosynthesis Enzymes, Are Essential for Tumorigenesis and In Planta Growth by P. syringae pv. savastanoi NCPPB 3335
Source: Front Plant Sci. 2020 Aug 21;11:1294. doi: 10.3389/fpls.2020.01294 (PMC7472798; doi:10.3389/fpls.2020.01294)
Supplement: Supplementary file 8 [file DataSheet_8.pdf]

Table S2. List and application of primers used in this work

Primer	Sequence (5'-3')[a]	5´ position[b]	Remarks or reference
**Construction of mutants and complementation**			
ExpptZ-F1	AATATGCTGAGCCTGGCAGA	29513*	Cloning of gene *ptz* in pBBR1MCS-5
ExpptZ-R2	CCTATGCCGATTGAAAGCAT	28142	
FAnewptz-F	GGCACATTCTGCTGAATG	30134*	Cloning the upstream flanking region of gene *ptz* for deletion by marker exchange
FAnewptz-R	CCCTATAGTGAGTCGGATCCCATCC GCACCTCCTAATC	28943	
FP_ptz-F18	GGATCCGACTCACTATAGGGTAAG GAGGATAAGACTGC	28245*	Cloning the downstream flanking region of gene *ptz* for deletion by marker exchange
FP_ptz-R1200	CATCAAGCTGACGCTATG	27023	
iptC_F	AGCGCTTGAGCTTTTGACTG	17895	Cloning of gene *idi* in pME6031
iptC_R	CAGAAATCTGCCCGAGAAAC	19011*	
iptSAM_F	TTTTGCCAAATCAGGAAGTC	16701	With primer iptC_R, cloning of gene *idi* with the upstream CDS PSPSV_C0023
mutipt_F	AGATTCATCGTTCGAGGCCC	16836	Cloning of a 3394 nt fragment for the deletion of *idi* by marker exchange
mutipt_R	CCTTCCTGAGTACGAGTCGC	20229*	
ptz_F	AAGACCCATACTGGTGCGATAG	28623*	To check presence of *ptz*
ptz_R	CCGCAACCAACTCGTCTAAC	28471	
ptz_F1	GAATTCAATATGCTGAGCCTGGCAGA	29513*	Cloning of gene *ptz* in pME6031
ptz_R1	GAATTCCCTATGCCGATTGAAAGCAT	28142	
**Conservation of *ptz-idi***			

idi_F	GGGATGGCTGAGAAGATTGA	18521	To check presence of *idi*
idi_R	TGGATTTCGGAAACCTCATC	18882	
ptz_A1F	AATCGTCGCATTTCTGAAGG	28759*	To check presence of allele *ptz*1
ptz_A1R	CTACGTTCAGCGAGCCACTC	28251	
ptz_A2F	TTTTGGACCTACGTGCTGTG		To check presence of allele *ptz*2
ptz_A2R	ATATCCGGGTCCAATTCTTG		
ptz2SAMidi_F	ATACCGGCCACTTGGAAAG		To check presence of allele *ptz*2, PSPSV_C0023 and *idi*
ptz2SAMidi_R	CAGGCGTCCGTCTTTGTC		
**Other purposes**			
iaaM_F	TCGGGTAGCGATAGTCGGTG	1092518*	To check presence of *iaaM*
iaaM_R	TTGCATGGCCCGATTACTGC	1091630	
IS50_F	ACACAGATTTAGCCCAGTCG		Mapping of Tn*5*-GDYN1 insertions
Km R-768	TTGCATCAGCCATGATGG		Plasmid sequencing
Oligo P1	GTGTAGGCTGGAGCTGCTTC		(Zumaquero et al., 2010)
SEQ	GTAAAACGACGGCCAGTG		Universal primer
SP6	ATTTAGGTGACACTATAG		Universal primer

[a] Restriction sites added to the primers to facilitate cloning are underlined.

[b] Indicates the coordinates of the annealing point of the first nucleotide of each primer in pPsv48A (accession no. FR820585), pPsv48C (accession no. FR820587) or NCPPB3335 (accession no. NZ_CP008742). Asterisks indicate that the primer anneals in the reverse strand of the DNA molecule.

Zumaquero, A., Macho, A.P., Rufian, J.S., and Beuzon, C.R. (2010). Analysis of the role of the type III effector inventory of *Pseudomonas syringae* pv. phaseolicola 1448A in interaction with the plant. *Journal of Bacteriology* 192(17), 4474-4488. doi: 10.1128/jb.00260-10.
