# Supplementary material for: Genes ptz and idi, Coding for Cytokinin Biosynthesis Enzymes, Are Essential for Tumorigenesis and In Planta Growth by P. syringae pv. savastanoi NCPPB 3335
Source: Front Plant Sci. 2020 Aug 21;11:1294. doi: 10.3389/fpls.2020.01294 (PMC7472798; doi:10.3389/fpls.2020.01294)
Supplement: Supplementary file 9 [file DataSheet_9.pdf]

**Table S3.** Production of cytokinins in minimal medium by derivatives of *Pseudomonas syringae* pv. savastanoi NCPPB 3335 cured of different native plasmids or mutated in genes *ptz* and *idi*, and complemented strains

| | **Amount of cytokinins in culture filtrates (nM) per strain**[a] | | | | | | | | | |
|---|---|---|---|---|---|---|---|---|---|---|
| | | | | | **Δ*ptz*** | | **Δ*idi*$^{-}$** | | **Δ*ptz*$^{-}$ *idi*$^{-}$** | |
| | **WT**[b] | **ΔAB** | **ΔC** | **ΔABC** | **-** | **+*ptz*** | **-** | **+*idi*** | **-** | **+*ptz* +*idi*** |
| **Precursors** | | | | | | | | | | |
| **iPRMP**[c] | 0.2 ± 0.0 | 0.2 ± 0.0 | 0.2 ± 0.0 | 0.2 ± 0.0 | 0.1 ± 0.0 | 0.4 ± 0.0 | 0.2 ± 0.0 | 0.2 ± 0.0 | 0.1 ± 0.0 | 0.2 ± 0.0 |
| **tZRMP** | 7.7 ± 0.8 | ≤ 0.05 | 3.8 ± 0.5 | ≤ 0.05 | ≤ 0.05 | 38.6 ± 4.6 | 9.5 ± 0.8 | 9.2 ± 1.0 | ≤ 0.05 | 7.3 ± 0.4 |
| **DHZMP** | ≤ 0.05 | *<LOD* | ≤ 0.05 | *<LOD* | *<LOD* | ≤ 0.05 | ≤ 0.05 | ≤ 0.05 | *<LOD* | ≤ 0.05 |
| **∑ (%)** | 8.0 ± 0.8 | 0.2 ± 0.0 | 4.0 ± 0.5 | 0.2 ± 0.0 | 0.2 ± 0.0 | 39.1 ± 4.6 | 9.7 ± 0.8 | 9.4 ± 1.0 | 0.2 ± 0.0 | 7.6 ± 0.4 |
| **Transporters** | | | | | | | | | | |
| **iPR** | 0.1 ± 0.0 | ≤ 0.05 | 0.1 ± 0.0 | 0.1 ± 0.0 | ≤ 0.05 | 0.2 ± 0.0 | 0.1 ± 0.0 | 0.4 ± 0.1 | ≤ 0.05 | 0.1 ± 0.0 |
| **tZR** | 9.9 ± 1.0 | ≤ 0.05 | 14.4 ± 3.2 | ≤ 0.05 | ≤ 0.05 | 79.0 ± 6.9 | 12.8 ± 1.0 | 29.0 ± 3.0 | ≤ 0.05 | 13.1 ± 1.4 |
| **DHZR** | 0.3 ± 0.0 | ≤ 0.05 | 0.6 ± 0.2 | ≤ 0.05 | ≤ 0.05 | 2.0 ± 0.3 | 0.4 ± 0.0 | 0.8 ± 0.1 | ≤ 0.05 | 0.3 ± 0.0 |
| **∑ (%)** | 10.2 ± 1.0 | ≤ 0.05 | 15.1 ± 3.4 | 0.1 ± 0.0 | ≤ 0.05 | 81.2 ± 7.2 | 13.2 ± 1.0 | 30.1 ± 3.1 | ≤ 0.05 | 13.6 ± 1.4 |
| **Active forms** | | | | | | | | | | |
| **iP** | 3.4 ± 0.5 | 1.2 ± 0.2 | 5.6 ± 0.3 | 1.7 ± 0.2 | 0.9 ± 0.1 | 10.0 ± 1.7 | 3.9 ± 0.4 | 3.6 ± 0.4 | 1.6 ± 0.3 | 3.1 ± 0.4 |
| **tZ** | 66.1 ± 5.5 | ≤ 0.05 | 107.3 ± 8.3 | ≤ 0.05 | ≤ 0.05 | 253.9 ± 13.7 | 101.7 ± 8.0 | 101.4 ± 8.3 | 0.1 ± 0.0 | 80.6 ± 5.1 |
| **DHZ** | 4.9 ± 0.5 | ≤ 0.05 | 10.2 ± 1.2 | ≤ 0.05 | ≤ 0.05 | 42.1 ± 5.8 | 8.2 ± 0.9 | 7.3 ± 1.0 | ≤ 0.05 | 4.8 ± 0.4 |
| **∑ (%)** | 74.5 ± 6.4 | 1.3 ± 0.2 | 123.0 ± 9.8 | 1.8 ± 0.2 | 0.9 ± 0.1 | 306.0 ± 21.2 | 113.8 ± 9.4 | 112.3 ± 9.7 | 1.7 ± 0.3 | 88.5 ± 5.9 |

| | | | | | | | | | | |
|---|---|---|---|---|---|---|---|---|---|---|
| **Glycosilated (inactive forms)** | | | | | | | | | | |
| **tZ9G** | 0.3 ± 0.0 | - | 0.4 ± 0.0 | ≤ 0.05 | ≤ 0.05 | 1.3 ± 0.2 | 0.4 ± 0.0 | 0.4 ± 0.0 | ≤ 0.05 | 0.3 ± 0.0 |
| **DHZ9G** | ≤ 0.05 | ≤ 0.05 | ≤ 0.05 | ≤ 0.05 | ≤ 0.05 | 0.2 ± 0.0 | ≤ 0.05 | ≤ 0.05 | ≤ 0.05 | ≤ 0.05 |
| **tZOG** | 0.1 ± 0.0 | *<LOD* | 0.1 ± 0.0 | *<LOD* | *<LOD* | 0.3 ± 0.1 | ≤ 0.05 | 0.1 ± 0.0 | *<LOD* | ≤ 0.05 |
| **DHZOG** | ≤ 0.05 | ≤ 0.05 | ≤ 0.05 | ≤ 0.05 | ≤ 0.05 | ≤ 0.05 | ≤ 0.05 | ≤ 0.05 | ≤ 0.05 | ≤ 0.05 |
| **∑ (%)** | 0.4 ± 0.0 | ≤ 0.05 | 0.6 ± 0.1 | ≤ 0.05 | ≤ 0.05 | 1.8 ± 0.3 | 0.4 ± 0.0 | 0.5 ± 0.0 | ≤ 0.05 | 0.4 ± 0.0 |
| **Total** | | | | | | | | | | |
| **∑ (%)** | 93.0 ± 8.3 | 1.5 ± 0.2 | 142.6 ± 13.7 | 2.0 ± 0.3 | 1.1 ± 1.1 | 428.0 ± 33.3 | 137.2 ± 11.2 | 152.3 ± 13.8 | 1.9 ± 0.3 | 110.1 ± 7.8 |
| **cZRMP** | 0.2 ± 0.0 | 0.2 ± 0.0 | 0.1 ± 0.0 | 0.2 ± 0.0 | 0.1 ± 0.0 | 0.3 ± 0.0 | 0.1 ± 0.0 | 0.1 ± 0.0 | 0.1 ± 0.0 | 0.1 ± 0.0 |
| **cZR** | 0.1 ± 0.0 | ≤ 0.05 | 0.1 ± 0.0 | 0.1 ± 0.0 | ≤ 0.05 | 0.2 ± 0.0 | ≤ 0.05 | 0.1 ± 0.0 | ≤ 0.05 | 0.1 ± 0.0 |
| **cZ** | 0.1 ± 0.0 | 0.1 ± 0.0 | 0.2 ± 0.0 | 0.1 ± 0.0 | 0.1 ± 0.0 | 0.4 ± 0.0 | 0.2 ± 0.0 | 0.1 ± 0.0 | 0.1 ± 0.0 | 0.1 ± 0.0 |
| **cZOG** | *<LOD* | *<LOD* | *<LOD* | *<LOD* | *<LOD* | *<LOD* | *<LOD* | *<LOD* | *<LOD* | *<LOD* |
| **∑ (%)** | 0.4 ± 0.0 | 0.4 ± 0.0 | 0.4 ± 0.0 | 0.3 ± 0.0 | 0.2 ± 0.0 | 0.9 ± 0.0 | 0.3 ± 0.0 | 0.3 ± 0.0 | 0.3 ± 0.0 | 0.2 ± 0.0 |

[a] Figures represent the average of four biological replicates with two technical replicates each (N=8) in fmol/μL ± SE rounded to one decimal. Total is the sum of precursors, glycosylated (inactive) and active forms.

[b] All strains derive from the wild type strain NCPPB 3335 (WT) and are summarized in Table S1. Names indicate the plasmids or genes that are missing in each strain; +*ptz* and +*idi* denote complementation with a cloned wild type copy of genes *ptz* and/or *idi*.

[c] Abbreviations: *cis*- and *trans*-zeatin (cZ and tZ), dihydrozeatin (DHZ), and isopentenyl adenine (iP), with the following suffixes, 9G, 9-glucoside; MP, monophosphate; OG, *O*-glucoside; R, riboside. <LOD, less than the limit of detection.
